# Influence of Ion Diffusion on the Lithium–Oxygen Electrochemical Process and Battery Application Using Carbon Nanotubes–Graphene Substrate

**DOI:** 10.1021/acsami.3c05240

**Published:** 2023-08-08

**Authors:** Stanislav Levchenko, Vittorio Marangon, Sebastiano Bellani, Lea Pasquale, Francesco Bonaccorso, Vittorio Pellegrini, Jusef Hassoun

**Affiliations:** † Department of Chemical, Pharmaceutical and Agricultural Sciences, University of Ferrara, Via Fossato di Mortara 17, Ferrara 44121, Italy; ‡ Graphene Labs, Istituto Italiano di Tecnologia, Via Morego 30, Genoa 16163, Italy; § BeDimensional S.p.A., Lungotorrente Secca 30r, 16163 Genoa, Italy; ∥ Materials Characterization Facility, Istituto Italiano di Tecnologia, Via Morego 30, Genova 16163, Italy; ⊥ National Interuniversity Consortium of Materials Science and Technology (INSTM), University of Ferrara Research Unit, Via Fossato di Mortara, 17, 44121 Ferrara, Italy

**Keywords:** Li−O_2_ battery, diffusion, cycle life, MWCNTs, few-layer graphene, energy storage

## Abstract

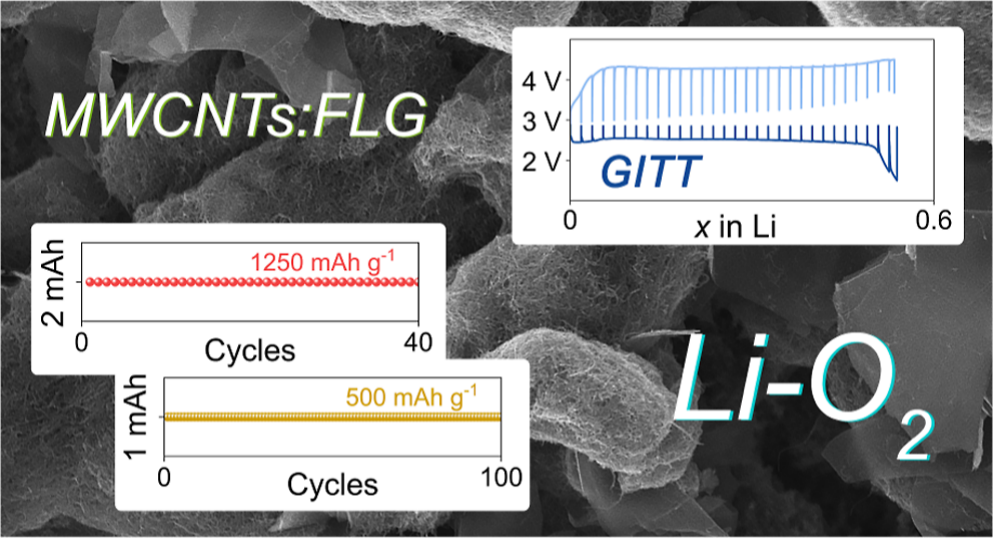

Lithium–oxygen (Li–O_2_) batteries are nowadays among the most appealing next-generation energy storage systems in view of a high theoretical capacity and the use of transition-metal-free cathodes. Nevertheless, the practical application of these batteries is still hindered by limited understanding of the relationships between cell components and performances. In this work, we investigate a Li–O_2_ battery by originally screening different gas diffusion layers (GDLs) characterized by low specific surface area (<40 m^2^ g^–1^) with relatively large pores (absence of micropores), graphitic character, and the presence of a fraction of the hydrophobic PTFE polymer on their surface (<20 wt %). The electrochemical characterization of Li–O_2_ cells using bare GDLs as the support indicates that the oxygen reduction reaction (ORR) occurs at potentials below 2.8 V vs Li^+^/Li, while the oxygen evolution reaction (OER) takes place at potentials higher than 3.6 V vs Li^+^/Li. Furthermore, the relatively high impedance of the Li–O_2_ cells at the pristine state remarkably decreases upon electrochemical activation achieved by voltammetry. The Li–O_2_ cells deliver high reversible capacities, ranging from ∼6 to ∼8 mA h cm^–2^ (referred to the geometric area of the GDLs). The Li–O_2_ battery performances are rationalized by the investigation of a practical Li^+^ diffusion coefficient (*D*) within the cell configuration adopted herein. The study reveals that *D* is higher during ORR than during OER, with values depending on the characteristics of the GDL and on the cell state of charge. Overall, *D* values range from ∼10^–10^ to ∼10^–8^ cm^2^ s^–1^ during the ORR and ∼10^–17^ to ∼10^–11^ cm^2^ s^–1^ during the OER. The most performing GDL is used as the support for the deposition of a substrate formed by few-layer graphene and multiwalled carbon nanotubes to improve the reaction in a Li–O_2_ cell operating with a maximum specific capacity of 1250 mA h g^–1^ (1 mA h cm^–2^) at a current density of 0.33 mA cm^–2^. XPS on the electrode tested in our Li–O_2_ cell setup suggests the formation of a stable solid electrolyte interphase at the surface which extends the cycle life.

## Introduction

The impellent need for efficient energy storage to stabilize the renewable power grids and provide satisfactory autonomy to electronic devices, including electric vehicles, has triggered a relevant breakthrough in the field of rechargeable batteries.^1,2^ Moreover, excessive ambient pollution and anomalously fast climate change during the recent years have focused the research efforts on developing sustainable technologies that can effectively replace Li-ion batteries based on critical and expensive raw materials, e.g., Co, Ni, and Mn.^3^ Among the various electrochemical energy storage systems, lithium–sulfur (Li–S) and Li–O_2_ batteries rely on abundant cathode materials, limiting their environmental and economic impact compared to Li-ion batteries.^4−6^ Furthermore, Li can electrochemically react with either S or O_2_ according to conversion processes involving multiple electrons/ions exchange, leading to practical energy densities above 500 W h kg^–1^, outperforming the state-of-the-art Li-ion batteries based on Li^+^-insertion-type electrodes.^7,8^ Particular interest has been devoted to rechargeable Li–O_2_ batteries operating in organic solvents because of their notable energy density (i.e., ∼3400 W h kg^–1^ for the schematic reaction Li_2_O_2_ ⇄ 2Li + O_2_) and potentially low life cycle environmental burdens.^5,9^ A relevant boost to these intriguing systems has been achieved by the use of ad hoc-designed electrolytes, including those based on glymes with the general formula CH_3_O(CH_2_CH_2_O)_*n*_CH_3_ characterized by chemical and electrochemical stabilities, as well as by limited cost and low toxicity.^10,11^ In particular, glymes with sufficiently long chains and low volatility can form in Li–O_2_ batteries stable coordination complexes with the reactive peroxide and superoxide radicals during ORR,^12,13^ and can withstand oxidation at potential as high as 4.8 V vs Li^+^/Li upon OER.^6^ The effect of the Li salt nature and concentration on the operation of the Li–O_2_ cell has been investigated by several studies, reporting promising results for cells using lithium trifluoromethanesulfonate (LiCF_3_SO_3_) and lithium bis(trifluoromethanesulfonyl)imide (LiTFSI) in glyme-based electrolytes characterized by high Li^+^ transference number and ionic conductivity, e.g., with tetraethylene glycol dimethyl ether (TEGDME) as the solvent.^6,14,15^ Despite the role of the Li^+^ diffusion to the electrode–electrolyte interphase on the cell performances has been widely investigated for Li-ion^16−19^ and Li–S batteries,^20,21^ only a limited deal of studies correlated the kinetics of Li^+^ diffusion to the performances of Li–O_2_ batteries.^22^ Efficient ORR/OER processes have been suggested for Li–O_2_ cells using GDLs, for facilitating the diffusion of involved species, with various substrates which promote the reaction kinetics, e.g., nanosized carbon,^14,23,24^ metals,^25−28^ metal oxides,^29−31^ and conductive polymers.^32^ Based on these premises, herein we reported a detailed study of various commercially available GDLs used as the support for the cathode material. We in-depth investigated the effects of the Li^+^ diffusion on the electrochemical process of Li–O_2_ batteries using these GDLs, which are characterized by different morphological and structural characteristics, as determined through scanning electron microscopy (SEM), X-ray diffraction (XRD), N_2_ physisorption measurements, and thermogravimetric analysis (TGA). The ORR and OER were examined through cyclic voltammetry (CV) measurements, while the evolution of the electrode/electrolyte interphase was monitored through electrochemical impedance spectroscopy (EIS) measurements. The diffusion kinetics were studied with galvanostatic intermittent titration technique (GITT), identifying the most suitable GDL to be combined with few-layer graphene (FLG) flakes and multiwalled carbon nanotubes (MWCNTs) for further improving the process in Li–O_2_ cells. MWCNTs have been chosen due to their optimal morphology that triggers an extremely reversible electrochemical process,^14^ while FLG flakes have been selected since they strongly enhance the stability of the MWCNT film on the GDL, improve the surface characteristics, and avoid cracks, thus increasing the cycle life of the cell. The identification of the correlation between electrode properties, Li^+^ diffusion kinetics, and cell performances is here proposed as an effective approach to design efficient and high-energy density Li–O_2_ batteries for practical applications.

## Experimental Section

### Material Characterization

Gas diffusion layers (GDL Sigracet Ion Power), referred to as 22BB, 28BC, 36BB, and 39BB, bare MWCNTs (>90% carbon basis, *D* × *L*: 110–170 nm × 5–9 μm, Sigma-Aldrich), and FLG produced by wet-jet mill (WJM) method (BeDimensional S.p.A.)^33^ were characterized by SEM, XRD, and TGA measurements. SEM images were acquired with a Zeiss EVO 40 microscope using back-scattered electrons and secondary electrons modes, while the corresponding EDS elemental mapping was recorded with a X-ACT Cambridge Instruments analyzer coupled to the SEM equipment. The XRD patterns of the GDLs were collected through a Bruker D8 Advance using a Cu Kα source (8.05 keV) by performing scans over the 2θ range between 10 and 60° with a step size of 0.02° and a rate of 10 s per step. The TGA measurements of the GDLs were carried out in the 25–1000 °C temperature range under N_2_ flow with a rate of 5 °C min^–1^, using a TGA 2 Mettler-Toledo instrument. The specific surface area and the porosity of the GDLs were determined by N_2_ adsorption at 77 K with an automated gas sorption analyzer (AutoSorb iQ, Quantachrome Instruments, USA). The samples were degassed under vacuum conditions at 150 °C overnight before each measurement. Specific surface area was calculated using the multi-point Brunauer–Emmett–Teller (BET) method,^34^ considering equally spaced points in a relative pressure range *P*/*P*_0_ from 0.05 to 0.30 with a correlation coefficient of above 0.999. The total pore volume was directly calculated from the volume of N_2_ held at the highest relative pressure (*P*/*P*_0_ = 0.99). The non-local density functional theory (NLDFT, implemented into Quantachrome’s data reduction software)^35^ was applied to the gas adsorption data using a slit-shape model to describe the pore-size distributions (PSDs) of the samples.

### Assembly of Li–O_2_ Cells and Electrochemical Tests

Foils of GDLs were cut into 16 mm diameter discs (geometric area: 2.0 cm^2^, mass: 13.4 mg for 22BB, 19.6 mg for 28BC, 17.6 mg for 36BB, and 18.2 mg for 39BB) and dried at 110 °C under vacuum for 3 h before transfer in an Ar-filled glovebox (MBraun) with H_2_O and O_2_ levels lower than 1 ppm. Top-meshed CR2032 coin-type cells (MTI Crop.) were assembled under an Ar atmosphere by stacking a GDL disc, a glass fiber Whatman GF/B separator with a diameter of 18 mm soaked with an excess (ca. 200 μL) of the electrolyte solution, and a Li disc with a diameter of 14 mm as the counter electrode. This two-electrode setup may have additional polarization compared to possible three-electrode configuration, in particular in view of Li reactivity. However, the above cell (i.e., top-meshed CR2032 coin cell) represents the most diffused system for practical Li–O_2_ battery characterization.^36^ Subsequently, the cells were inserted in sealed glass chambers and filled with pure oxygen to achieve the Li–O_2_ system. The electrolyte solution consisted of TEGDME (≥99%, Sigma-Aldrich) dissolving LiCF_3_SO_3_ (99.995% trace metals basis, Sigma-Aldrich) conductive salt with a concentration of 1 mol kg_solvent_^–1^. Before electrolyte preparation, TEGDME was kept in Ar-filled glovebox under molecular sieves (3 Å, rod, size 1/16 in., Honeywell Fluka) previously dried under vacuum at 280 °C for 5 days, until a water content lower than 10 ppm was verified by a 899 Karl Fischer Coulometer (Metrohm), while LiCF_3_SO_3_ salt was dried under vacuum for 2 days at 110 °C. The electrochemical characterization of Li–O_2_ cells was carried out by means of CV and EIS measurements using a VersaSTAT MC Princeton Applied Research (PAR) potentiostat/galvanostat. The CV measurements consisted of three subsequent potential scans between 2.5 and 4.2 V vs Li^+^/Li at 0.05 mV s^–1^, while EIS spectra of the cells were recorded at the open-circuit voltage (OCV) condition and after each voltammetry cycle. Additional CV–EIS measurements were run on Li–O_2_ cells using a CV potential range of 1.5–4.3 V vs Li^+^/Li with a scan rate of 0.05 mV s^–1^ and performing EIS at the OCV condition and after each voltammetry cycle. All EIS spectra were recorded through an AC voltage signal with an amplitude of 10 mV in the 500 kHz to 100 mHz frequency range. The spectra were subsequently fitted by an equivalent electrical circuit model using the non-linear least squares (NLLS) method through Boukamp software.^37,38^ Only fits with a chi-square (χ^2^) value of the order of 10^–4^ or lower were considered. EIS measurements were also conducted on symmetrical Li–Li and GDL(39BB)-GDL(39BB) cells in an O_2_ atmosphere at the OCV condition in the 500 kHz to 100 mHz frequency range with AC voltage signal with an amplitude of 10 mV. Polarization curves were recorded through galvanodynamic reduction scans between 0 and −20 mA on either a Li–Li and Li-GDL(39BB) cells in an O_2_ atmosphere using a step height of 0.1 mA and a step time of 10 s. Galvanostatic charge/discharge cycling measurements were carried out on Li–O_2_ cells using the various GDLs by applying a current of 0.2 mA (0.1 mA cm^–2^ considering the geometric area of the GDL discs of 2.0 cm^2^) and limiting the cell capacity to 2 mA h, or by setting the cell voltage between 1.5 and 4.5 V (without any capacity limitation). The GITT measurements were performed to record the potential of Li–O_2_ cells with the various GDLs over the exchanged lithium equivalents (*x*) in the 1.5–4.5 V vs Li^+^/Li range, using square current pulses of 0.4 mA for 1 h followed by potential relaxation steps of 1 h at the reached state of charge (SOC). An additional Li–O_2_ cell was assembled using the GDL 39BB coated with MWCNTs and FLG. The latter were deposited onto the GDL by Doctor Blade (MTI Corp.) casting of a slurry composed by 80 wt % of MWCNTs, 10 wt % of FLG, and 10 wt % of polyvinylidene fluoride (PVDF 6020 Solef) dispersed in *N*-methyl-2-pyrrolidone (NMP, Sigma-Aldrich). The electrode tape was dried at 70 °C, cut into 16 mm-diameter discs (geometric area: 2.0 cm^2^), and dried at 110 °C under vacuum for 3 h before transfer in Ar-filled glovebox. The final mass loading of MWCNTs/FLG on the GDL support ranged from 0.8 to 1.0 mg cm^–2^. Galvanostatic charge/discharge measurements were carried out on this Li–O_2_ cell by applying a current rate of 0.66 mA (0.33 mA cm^–2^) and limiting the cell capacity to 2 mA h (1 mA h cm^–2^) and 1 mA h (0.5 mA h cm^–2^) in the 1.5–4.8 V voltage range. The charge/discharge galvanostatic tests and GITT were performed using a MACCOR series 4000 battery test system, and all the electrochemical tests were performed at 25 °C.

Galvanostatic and CV tests were carried out on cells using lithium discs with thickness of 250 μm and mass of about 20 mg, while Li–O_2_ cells for GITT measurements employed lithium anodes with thickness and mass limited to 70 μm and 7 mg, respectively. In addition, a 39BB GDL coated with MWCNTs/FLG (composite loading: 0.8 mg cm^–2^) was galvanostatically discharged and charged for three cycles in a Li–O_2_ cell at 0.66 mA with a capacity limited to 2 mA h between 1.5 and 4.8 V, and subsequently retrieved for XPS analysis. The XPS measurements were performed on the cycled electrode and on a pristine one for comparison with a Kratos Axis UltraDLD spectrometer, equipped with a monochromatic Al Kα source, operating at 20 mA and 15 kV. To prevent air contamination, the samples were moved from an Ar-filled glovebox to the XPS system using a hermetically sealed transfer chamber. Wide scans were carried out with an analysis area of 300 × 700 μm and a pass energy of 160 eV. High-resolution spectra were collected over the same analysis area at a pass energy of 20 eV. Spectra were charge-corrected to the C 1s peak at 284.5 eV for sp^2^ carbon (C=C) and were analyzed using CasaXPS software (version 2.3.25).

## Results and Discussion

### Morphology and Structure of the GDLs

**Figure 1:**
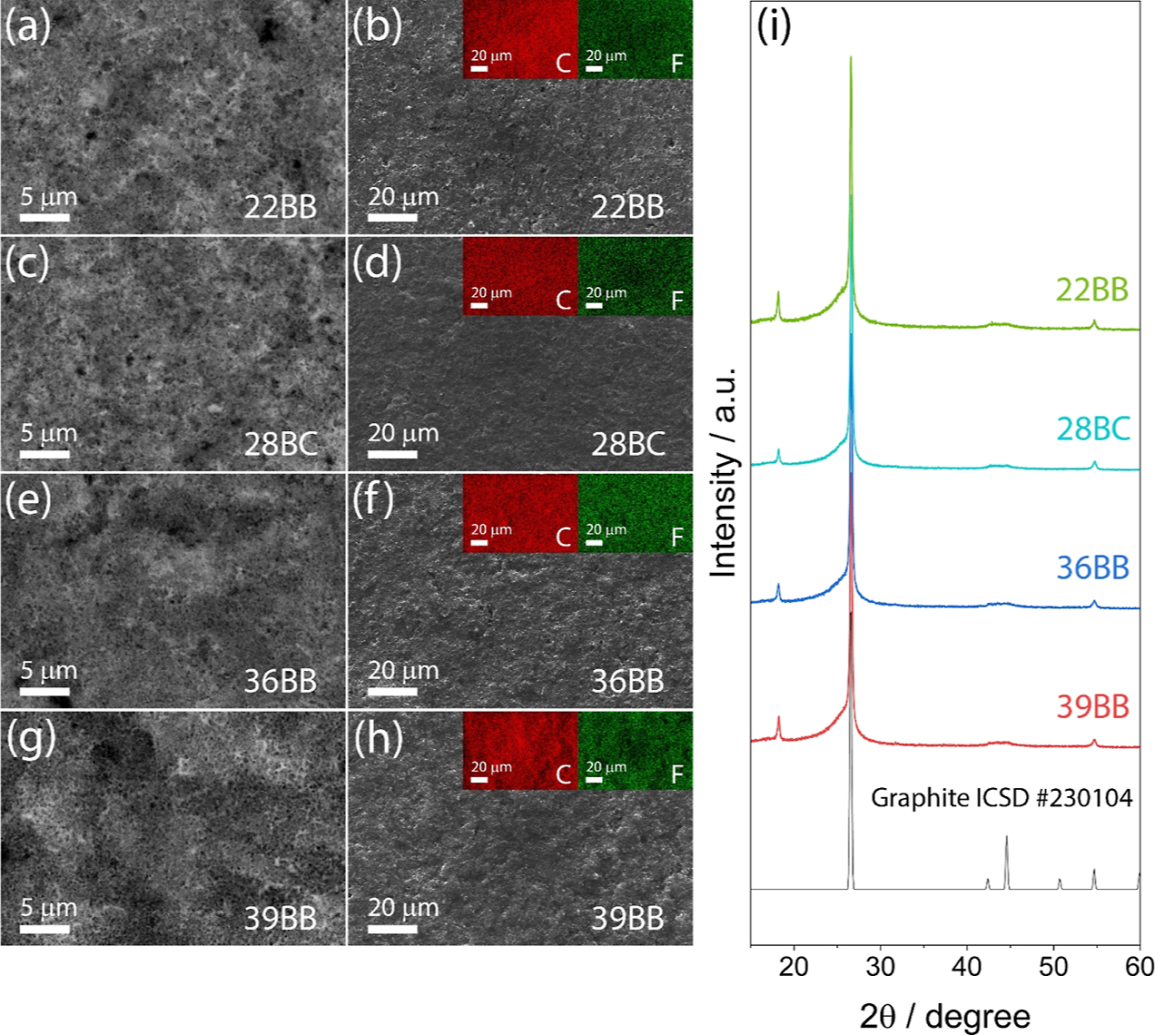
(a–h) SEM images of the (a,b) 22BB, (c,d) 28BC, (e,f) 36BB, and (g,h) 39BB GDLs acquired in either (a,c,e,g) back-scattered electrons or (b,d,f,h) secondary electrons mode; insets in panels (b,d,f,h) show the corresponding EDS elemental maps for C and F. (i) XRD patterns measured for 22BB (green), 28BC (cyan), 36BB (blue), and 39BB (red). The reference pattern for graphite (black, ICSD #230104) is also reported for comparison.

The morphological and structural characteristics of the 22BB, 28BC, 36BB, and 39BB GDLs are evaluated through SEM-EDS (Figure 1a–h) and XRD (Figure 1i) measurements, respectively. The SEM images acquired in the back-scattered electrons mode (Figure 1a,c,e,g) show the presence of sub-micron (nanometric) primary particles locally aggregated into secondary micrometric domains in all the GDLs. The aggregates in 22BB (Figure 1a) and 28BC (Figure 1c) appear smaller than those in 36BB (Figure 1e) and 39BB (Figure 1g), leading to a different surface morphology. The latter can be qualitatively evaluated from the secondary electron SEM images (Figure 1b,d,f,h, and images with higher magnification are reported in Figure S1 in Supporting Information). Accordingly, the 22BB and 28BC GDLs reveal smaller aggregates compared to 36BB and 39BB samples, in agreement with the experimental surface area discussed afterward. The EDS elemental mapping recorded on secondary electron SEM images (insets of Figure 1b,d,f,h) shows the presence of F in addition to that of C. The F signal is associated to the polytetrafluoroethylene (PTFE) binder, which is typically applied to the GDLs to improve their mechanical stability and hydrophobicity, however with an insulating character that may affect the reaction kinetics. Figure 1i shows the XRD patterns of the GDLs, which exhibit a main sharp peak at 2θ = 26.6° and a secondary signal at 2θ = 54.7° ascribed to the graphite,^39^ broad shoulders in the 20–30 and 40–45° 2θ ranges indicating the co-presence of amorphous carbon,^40^ and a peak at 2θ = 18° associated to the PTFE.^41^ It is worth mentioning that the difference between EDS and XRD responses is related with the nature of the two techniques. Indeed, EDS focuses mainly on the electrode surface and can detect species without any crystallinity, while XRD detects only crystalline species located into the whole electrode structure. Overall, SEM-EDS and XRD analyses reveal that all the GDLs are formed by both graphitic and amorphous carbons, linked with PTFE binder, and exhibit different surface morphologies which may therefore influence the electrochemical processes occurring in the Li–O_2_ battery.

**Figure 2:**
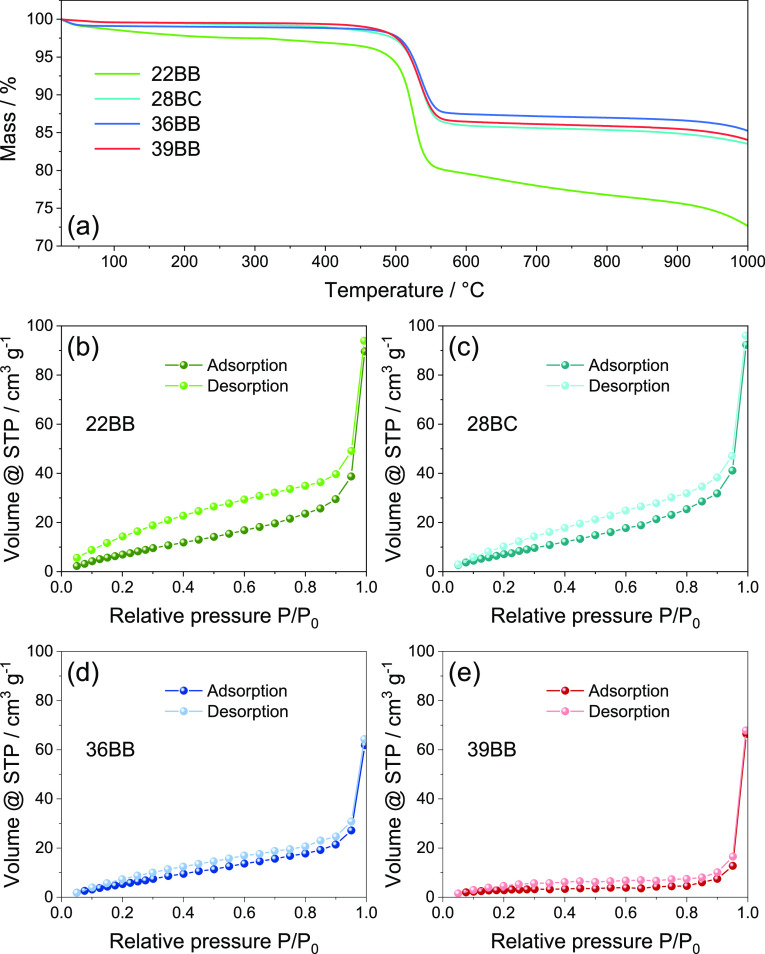
(a) TGA curves measured under N_2_ flow in the 25–1000 °C temperature range for 22BB (green), 28BC (cyan), 36BB (blue), and 39BB (red); (b–e) N_2_ absorption/desorption isotherms for (b) 22BB, (c) 28BC, (d) 36BB, and (e) 39BB, used to estimate the specific surface area and pore size characterization (see Table 1).

The GDLs are further evaluated through TGA performed under N_2_ to determine the binder content (Figure 2a), while N_2_ adsorption measurements at 77 K (Figure 2b–e and Table 1) are carried out to assess their surface area and PSD.^34^ The thermogravimetric curves (Figure 2a) and the corresponding differential thermogravimetry (DTG) curves (Figure S2, Supporting Information) show that the GDLs undergo a weight loss between 25 and 100 °C ascribed to the removal of absorbed water. The weight loss between 500 and 550 °C is associated to the PTFE decomposition,^42^ while the weight loss starting at 950 °C is attributed to the degradation of the carbonaceous structure of the GDLs. Importantly, the TGA data reveal that the GDLs have different contents of PTFE, i.e., 17 wt % for 22BB, 13 wt % for both 28BC and 39BB, and 12 wt % for 36BB. Moreover, 22BB exhibits the most pronounced weight loss below 200 °C, indicating a superior ability to absorb moisture compared to the other GDLs. Figure 2b–e shows the N_2_ adsorption/desorption isotherms for the various GDLs. Based on the International Union of Pure and Applied Chemistry (IUPAC) classification,^43^ all the isotherms can be classified as type II isotherms with a H3 hysteresis loop, indicating the presence of relatively large pores. Table 1 reports the compilation of textural parameters obtained after application of the BET equation and NLDFT method to the N_2_ adsorption data of the GDLs. The highest surface area of 39 m^2^ g^–1^ is found for 22BB, and the lowest one of 13 m^2^ g^–1^ for 39BB. 28BC and 36BB show intermediate BET surface area of 38 and 31 m^2^ g^–1^, respectively. The pore volumes are 0.14 cm^3^ g^–1^ for both 22BB and 28BC and 0.10 cm^3^ g^–1^ for 36BB and 39BB. The PSD analysis derived from the adsorption branch of the isotherms in Supporting Information (Figure S3) indicates two main populations of mesopores at ∼3 and 4.5 nm with intensities decreasing from 22BB to 28BC, 36BB, and 39BB. The minor peak centered at ∼30 nm shows similar intensity for all the GDLs.

**Table 1:** Data Derived from N_2_-Sorption Isotherms in Figure 2 for the GDLs

GDL	surface area [m^2^ g^–1^]	total pore volume (*P*/*P*_0_ = 0.99) [cm^3^ g^–1^]	average pore diameter [nm]
22BB	39	0.14	2.77
28BC	38	0.14	2.77
36BB	31	0.10	2.77
39BB	13	0.10	2.53

It is worth mentioning that the BET surface area detected herein may differ from the one fully accessible to the electroactive species which represents the electrochemically active surface. On the other hand, the BET surface area observed for the 22BB, 28BC, and 36BC GDLs is higher than that of the GDL 39BB. Therefore, the difference between the BET surface area observed herein between the GDLs may play a role in enhancing the cell performances of the materials in Li–O_2_ cells. Nevertheless, further discrepancies between the inter-fiber pores more readily accessible for Li_2_O_2_ formation compared to the mesopores of 3–4 nm diameter cannot be excluded, as suggested by literature studies.^44,45^ The 22BB and 28BC GDLs have similar surface area, while the TGA in Figure 2a shows that 22BB has a higher quantity of the PTFE binder (17%) compared to 28BC (13%). Hence, the higher ratio of the insulating polymer in 22BB compared to 28 BC may actually affect the CV curves, as demonstrated hereafter.

### Characteristics of the Li–O_2_ Electrochemical Process

**Figure 3:**
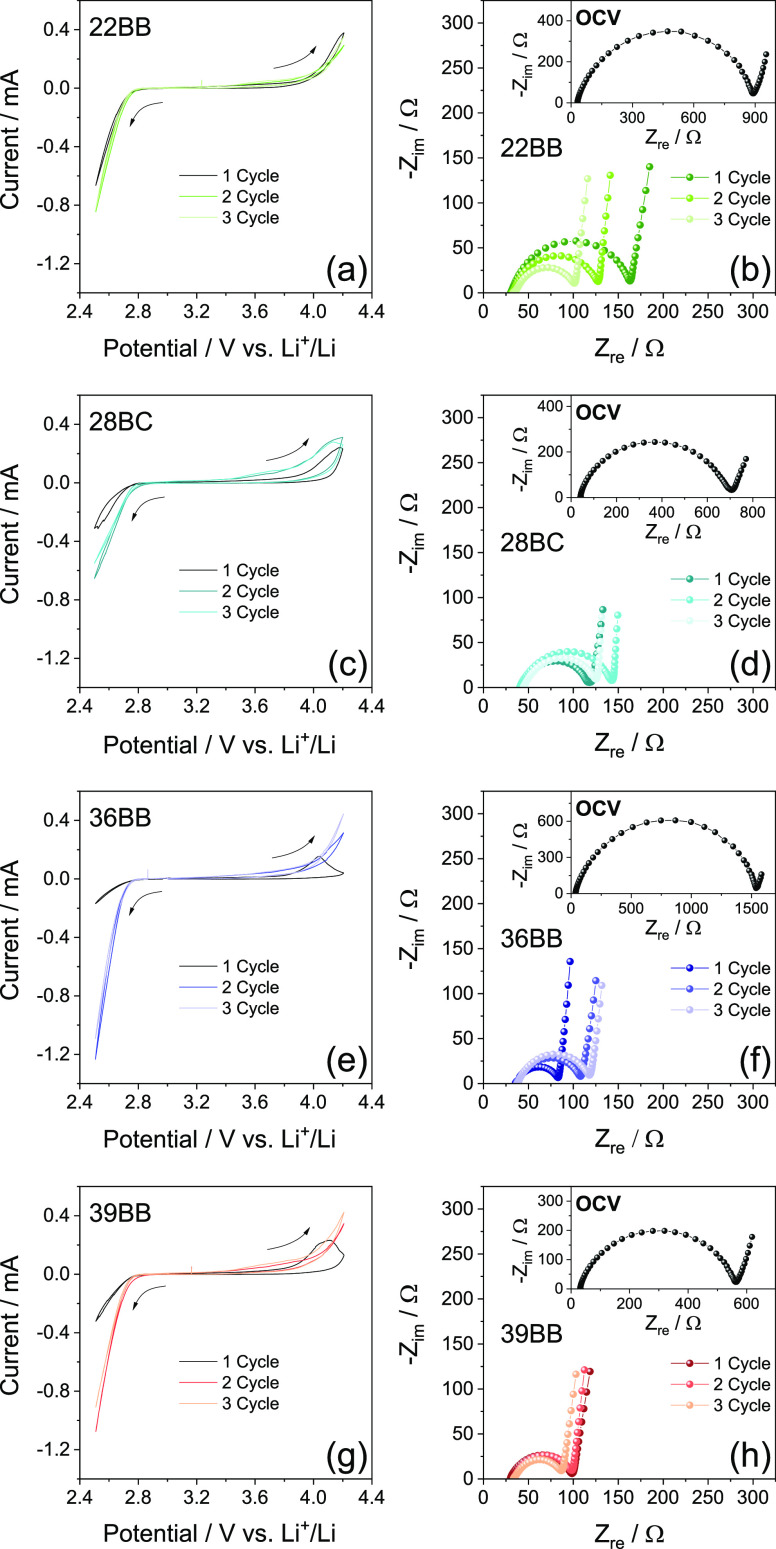
(a,c,e,g) CV curves and (b,d,f,h) Nyquist plots recorded before and after each CV cycle [see insets in panels (b,d,f,h) for OCV condition] measured for Li–O_2_ cells using (a,b) 22BB, (c,d) 28BC, (e,f) 36BB, or (g,h) 39BB as cathodes. CV potential range: 2.5–4.2 V vs Li^+^/Li; scan rate: 0.05 mV s^–1^. EIS frequency range: 500 kHz–100 mHz; alternate voltage signal: 10 mV.

The electrochemical behavior of the bare GDLs as cathodes in Li–O_2_ cells is studied through CV measurements, performed between 2.5 and 4.2 V vs Li^+^/Li (Figure 3a,c,e,g), and EIS measurements, carried out at the OCV condition and after each CV scan (Figure 3b,d,f,h). The potential window used for the CV favors the reversible redox process Li + 1/2O_2_ ⇄ 1/2Li_2_O_2_, which typically involves multiple steps and intermediates such as the lithium superoxide radical (LiO^•^_2_).^13^ The first CV curves measured for the cell using 22BB (Figure 3a), 28BC (Figure 3c), 36BB (Figure 3e), and 39BB (Figure 3g) reveal cathodic currents at potential lower than 2.8 V vs Li^+^/Li, which are attributed to the ORR, i.e., Li + 1/2O_2_ → 1/2Li_2_O_2_.^13^ The reverse oxidation steps, associated to the OER, i.e., Li_2_O_2_ → 2Li + O_2_, are instead revealed by the anodic currents at potentials exceeding 3.6 V vs Li^+^/Li.^13^ Interestingly, during the first CV cycle (black curves), the shape and intensity of the cathodic and anodic currents associated to the ORR and OER, respectively, appear to be influenced by the GDL characteristics. Indeed, the cells using 22BB (Figure 3a) show intense and narrow ORR and OER sharp current slopes rather than defined peaks. Instead, the cells using 28BC (Figure 3c), 36BB (Figure 3e), and 39BB (Figure 3g) reveal similar ORR current slope but with a lower intensity than 22BB, and OER reflecting broad peaks centered at ∼4.0 V vs Li^+^/Li. The higher ORR intensity of the cell using 22BB support with respect to the other GDLs may indicate a Li_2_O_2_ deposition initially triggered by its higher surface area (see Table 1). On the other hand, the formation of a defined OER peak in the cells using the 28BC, 36BB, and 39BB may account for the OER process promoted by a favorable morphology of the reaction products (Li_2_O_2_) due to the relevantly lower binder content in these GDLs compared to 22BB (see discussion of Figure 2).^14^ Despite the intensity of the CV peak does not directly account for the kinetics of the charge transfer, it may be associated with the various processes, including diffusion in the cell and reaction at the electrode/electrolyte interphase. Hence, the kinetics may be ascribed to the whole process, including ions and electrochemical species diffusion as well as charge transfer at the electrode/electrolyte interphase, in particular considering the geometry of the cell used herein to achieve the Li–O_2_ battery, that is, a top-meshed CR2032 coin cell.^36^ Furthermore, the use of a suitable three-electrode geometry in the Li–O_2_ cell may be hindered by the reactivity of the additional Li-reference electrode, and by possible leakage of the liquid electrolyte. Instead, the coin cell allows the study of the electrochemical reaction without the abovementioned issues, despite additional polarization due to the two-electrode configuration cannot be excluded. During the subsequent CV cycles, the cathodic current of the ORR increases for all GDLs, less remarkably for the cell using 22BB (Figure 3a) and more relevantly for the cells using 36BB (Figure 3e) and 39BB (Figure 3g), while the anodic current of the OER increases for all GDLs, except for 22BB. Furthermore, the OER CV shapes change for the cell using 36BB and 39BB from a broad but defined peak to a sloped profile. The increase of the cathodic currents during repeated CV cycles indicates the presence of an activation of the GDLs toward the ORR, instead the behavior of the anodic currents and related CV shapes during the OER appears more complex. The GDL activation toward ORR may be ascribed to the stabilization of the electrode/electrolyte region and the formation of a favorable SEI layer.^6^ Noteworthy, the activation process is particularly pronounced for the 36BB and 39BB GDLs, which are characterized by the lowest surface area and lowest porosity among the investigated samples (see Table 1). To elucidate the electrode/electrolyte interphase properties, EIS spectra of the Li–O_2_ cells are recorded before and after each CV cycle, as shown in Figure 3b,d,f,h for 22BB, 28BC, 36BB, and 39BB, respectively. The resulting Nyquist plots are fitted through the NLLS method, modeling the Li–O_2_ systems with a *R*_e_(*R*_1_*Q*_1_)*Q*_g_ equivalent circuit including resistive elements (*R*) and constant phase elements (*Q*), accounting for the electrolyte and the electrode/electrolyte interphase (see the top-side scheme in Figure S4 in Supporting Information).^37,38^ More in detail, *R*_e_ is the electrolyte resistance measured by the high-frequency intercept of the Nyquist plot; *R*_1_ and *Q*_1_, arranged in parallel in the (*R*_1_*Q*_1_) element, describe the processes related to the Li^+^ transfer and/or the SEI layer formation;^37,38^ the *R*_1_ resistance corresponds to the width of the semicircle in the high-medium frequency range;^37,38^ and lastly, *Q*_g_ is a constant phase element used to represent the low-frequency region of the Nyquist plot identifying the cell geometric capacitance and the diffusion-limited mass transport.^37,38^Table 2 shows the estimated parameters for the equivalent circuits of the investigated Li–O_2_ systems, as determined by the NLLS fitting. At OCV, the Li–O_2_ cells show high *R*_1_ with values ranging from 530 to ∼1520 Ω. After the first CV cycle, *R*_1_ significantly decreases to 135 Ω for 22BB (Figure 3b), 78 Ω for 28BC (Figure 3d), 49 Ω for 36BB (Figure 3f), and 69 Ω for 39BB (Figure 3h). After three CV cycles, *R*_1_ further decreases to 70 Ω for 22BB and to 55 Ω for 39BB, almost stabilizes at 83 Ω for 28BC, and increases to 82 Ω for 36BB (see Table 2).

**Table 2:** NLLS Analyses of the Nyquist Plots Recorded by EIS before and after Each CV Cycle (Potential between 2.5 and 4.2 V vs Li^+^/Li) for the Li–O_2_ Cells Using the Investigated GDLs as Cathodes^a^

GDL	cell condition	circuit	*R*_e_ [Ω]	*R*_1_ [Ω]	χ^2^
22BB	OCV	*R*_e_(*R*_1_*Q*_1_)*Q*_g_	30.1 ± 0.2	865 ± 6	6 × 10^–4^
	after 1 CV cycle	*R*_e_(*R*_1_*Q*_1_)*Q*_g_	31.1 ± 0.1	135 ± 1	4 × 10^–4^
	after 2 CVcycles	*R*_e_(*R*_1_*Q*_1_)*Q*_g_	32.7 ± 0.1	97.5 ± 0.8	4 × 10^–4^
	after 3 CVcycles	*R*_e_(*R*_1_*Q*_1_)*Q*_g_	34.6 ± 0.2	69.6 ± 0.9	5 × 10^–4^
28BC	OCV	*R*_e_(*R*_1_*Q*_1_)*Q*_g_	40.5 ± 0.3	647 ± 6	9 × 10^–4^
	after 1 CV cycle	*R*_e_(*R*_1_*Q*_1_)*Q*_g_	41.3 ± 0.2	77.6 ± 0.6	4 × 10^–4^
	after 2 CVcycles	*R*_e_(*R*_1_*Q*_1_)*Q*_g_	41.1 ± 0.2	104 ± 1	4 × 10^–4^
	after 3 CV cycles	*R*_e_(*R*_1_*Q*_1_)*Q*_g_	45.1 ± 0.1	82.5 ± 0.7	3 × 10^–4^
36BB	OCV	*R*_e_(*R*_1_*Q*_1_)*Q*_g_	36.0 ± 0.2	1516 ± 92	6 × 10^–4^
	after 1 CV cycle	*R*_e_(*R*_1_*Q*_1_)*Q*_g_	36.2 ± 0.2	48.7 ± 0.6	5 × 10^–4^
	after 2 CV cycles	*R*_e_(*R*_1_*Q*_1_)*Q*_g_	37.2 ± 0.1	72.8 ± 0.6	3 × 10^–4^
	after 3 CV cycles	*R*_e_(*R*_1_*Q*_1_)*Q*_g_	38.1 ± 0.1	82.1 ± 0.6	2 × 10^–4^
39BB	OCV	*R*_e_(*R*_1_*Q*_1_)*Q*_g_	30.7 ± 0.3	530 ± 4	8 × 10^–4^
	after 1 CV cycle	*R*_e_(*R*_1_*Q*_1_)*Q*_g_	30.4 ± 0.2	69.1 ± 0.6	4 × 10^–4^
	after 2 CV cycles	*R*_e_(*R*_1_*Q*_1_)*Q*_g_	33.9 ± 0.1	66.3 ± 0.6	4 × 10^–4^
	after 3 CV cycles	*R*_e_(*R*_1_*Q*_1_)*Q*_g_	34.1 ± 0.1	55.1 ± 0.7	4 × 10^–4^

a The NLLS fitting was performed with Boukamp software, and only χ^2^ values of the order of 10^–4^ or lower were accepted.^37,38^

In general, these EIS data confirm the cycling-induced activation of the electrode/electrolyte interphase for the ORR observed during CV, showing significant differences depending on morphological and structural characteristics of the investigated GDLs. In particular, after three CV cycles, the lowest *R*_e_ value is observed for 39BB, which has the lowest surface area and porosity among the GDLs. On the other hand, *R*_e_ remains almost constant after subsequent CV runs for all the GDLs, with values ranging between 30 and 45 Ω (Table 2). The trend observed for *R*_e_ indicates only minor electrolyte decomposition during cell operation.^46^

Additional EIS measurements are carried out on symmetric Li–Li and GDL(39BB)-GDL(39BB) cells, both assembled in an O_2_ atmosphere at the OCV condition (Figure S5 in Supporting Information). Figure S5a shows for the symmetric Li–Li cell the typical Nyquist plot including a semicircle at medium–high frequency ascribed to the electrode/electrolyte interphase, and a low frequency contribute related with the semi-finite Warburg-type Li^+^ diffusion. The cell shows a resistance around 100 Ω, that is much lower than that of the Li–O_2_ cell using the same GDL displayed in Figure 3h at the same condition (i.e., of about 600 Ω at the OCV) and comparable to the values achieved after cell cycling. This result supports the activation process experienced by the GDLs as the cell resistances in Figure 3h decrease to values comparable to that of the Li–Li cell upon CV, and actually suggests that the contribute of the Li electrode cannot be excluded in evaluating the Li–O_2_ cell impedance. On the other hand, the GDL(39BB)/GDL(39BB) cell (Figure S5b) shows a wide and noisy semicircle likely ascribed to possible side reaction of the electrolyte or ion diffusion, with a very large resistance value, i.e., extending 10,000 Ω, and suggesting the almost blocking character of this configuration due to the absence of the Li^+^ source in the electrodes.

**Figure 4:**
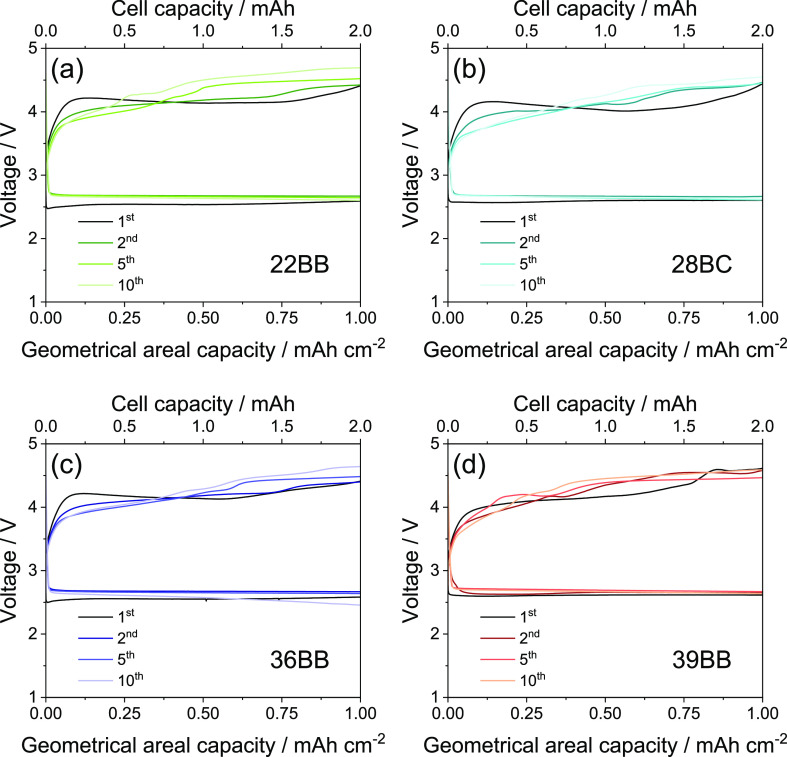
Voltage profiles during galvanostatic charge/discharge cycling measured for the Li–O_2_ cells using (a) 22BB, (b) 28BC, (c) 36BB, or (d) 39BB as cathodes, at a constant current of 0.2 mA and limiting the cell capacity to 2 mA h (1 mA h cm^–2^ considering the GDL geometric area of 2.0 cm^2^). Bottom *x*-axes report the geometrical areal capacity (mA h cm^–2^), while top *x*-axes show the cell capacity (mA h). Maximum voltage range: 1.5–4.8 V.

Figure 4 shows the galvanostatic charge/discharge curves measured for the Li–O_2_ cells using 22BB (Figure 4a), 28BC (Figure 4b), 36BB (Figure 4c), and 39BB (Figure 4d) as cathodes. The cells are cycled with a constant current of 0.2 mA, limiting the cell capacity at 2 mA h (1 mA h cm^–2^ considering the GDL geometric area of 2.0 cm^2^) that corresponds to charge and discharge processes of 10 h each. In addition, minimum and maximum voltage cutoff of 1.5 and 4.8 V, respectively, are used. This galvanostatic charge/discharge cycling procedure avoids excessive deposition of Li_2_O_2_ on the GDL surface and ensures reversible cell operation.^47^ The cell voltage profiles reveal the occurrence of the ORR and OER between 2.5 and 2.7 V and between 3.6 and 4.5 V, respectively. At the end of the first discharge/charge cycle, the Li–O_2_ cells exhibit similar polarizations (i.e., difference between the voltages achieved by the cell at the end of charge and at the end of discharge) of ∼1.8 V, except for the one using 39BB that show a polarization of ∼2.0 V likely due to the growth of larger insulating Li_2_O_2_ agglomerates.^15^ During subsequent charge/discharge cycles, all the investigated Li–O_2_ cells exhibit an activation for the ORR that occurs at slightly higher voltage, due to the abovementioned stabilization of the SEI upon the first charge/discharge cycle. After 10 cycles, the cells display different polarization values, i.e., 2.1 V for 22BB, 1.9 V for 28BC, 2.2 V for 36BB, and below 2.0 V for 39BB.

The difference between the voltages achieved by the cell at the end of charge and at the end of discharge is reported as a function of the cycle number in Figure S6 in Supporting Information, which shows the initial decrease of the polarization upon the above discussed GDL activation. After 2–3 cycles, the cell polarization increases for all the cells except that based on 39BB, for which the polarization starts to increase only after the 4th cycle and stabilizes at a final value (10th cycle) slightly lower than the initial one.^15^ Overall, these cell polarization trends indicate that 39BB is a particularly suitable GDL to ensure the formation of stable and effective electrode/electrolyte interphase for the realization of performant Li–O_2_ systems.

**Figure 5:**
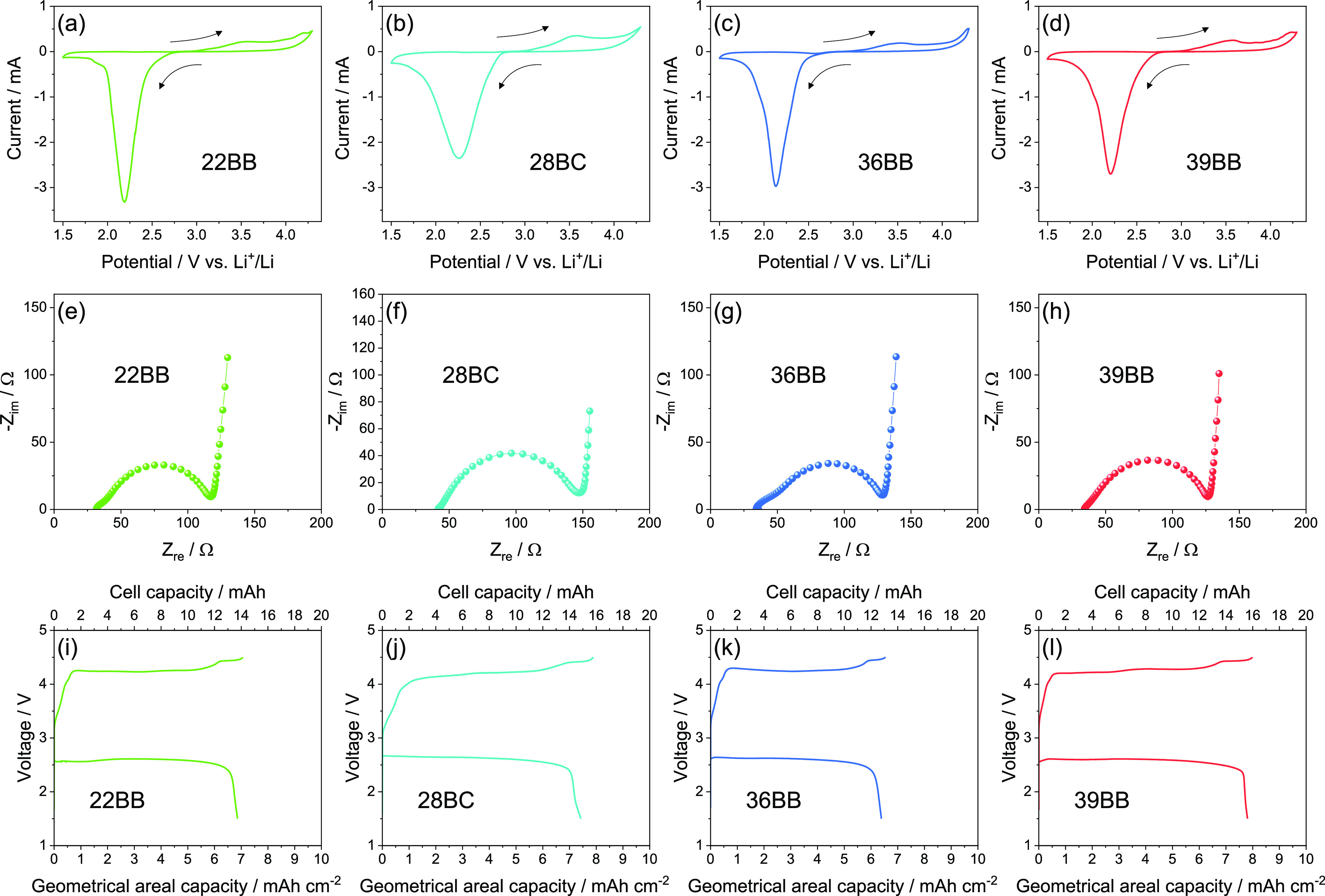
(a–d) CV curves, (e–h) Nyquist plots recorded by EIS, and (i–l) voltage profiles during galvanostatic charge/discharge cycling measured for the Li–O_2_ cells using (a,e,i) 22BB, (b,f,j) 28BC, (c,g,k) 36BB, or (d,h,l) 39BB as cathodes. CV potential range 1.5–4.3 V vs Li^+^/Li; scan rate: 0.05 mV s^–1^. EIS carried out after each CV scan in the 500 kHz to 100 mHz frequency range. Voltage profile measured for the investigated cells during galvanostatic charge/discharge cycling at 0.2 mA and voltage between 1.5 and 4.5 V with no cell capacity limitation; in panels (i–l), bottom *x*-axes report the geometrical areal capacity (mA h cm^–2^), while top *x*-axes show the cell capacity (mA h).

The GDLs are subsequently investigated by CV, EIS, and galvanostatic charge/discharge measurements using a wide potential range and without any capacity limitation. Previous paper suggested for the TEGDME-LiCF_3_SO_3_ solution and the PVDF binder anodic stability approaching 4.8 V,^36^ despite partial electrolyte oxidation during the OER at lower potentials,^48^ and side reaction due to the PVDF binder^49^ cannot be completely excluded. On the other hand, the reductive decomposition of the electrolyte typically occurs below 1 V, with formation of a stable passivation film at the electrode surface. Hence, the extended potential range is herein aimed to study of the effects of a massive Li_2_O_2_ deposition during cell discharge on kinetics, impedance, polarization, and maximum capacity of the investigated Li–O_2_ cells.^47,50,51^ Indeed, literature papers indicated that restricted potential ranges (e.g., 2.5–4.2 V vs Li^+^/Li in Figure 3) can allow the limitation of the undesired process, hold the high electrode conductivity, and increase the reversibility of the Li–O_2_ redox process in particular during ORR, instead the excessive Li_2_O_2_ electrodeposition achieved by voltammetry lowering the cathodic limit to 1.5 V vs Li^+^/Li can lead to a partial insulation of the electrode surface, which is reflected in a decrease of the reversibility.^14,36,47^Figure 5 displays the CV curves recorded in the 1.5–4.3 V vs Li^+^/Li potential window (Figure 5a–d), the EIS spectra acquired after each CV cycle (Figure 5e–h), and the cell voltage profiles measured during galvanostatic charge/discharge cycles at a constant current of 0.2 mA between 1.5 and 4.3 V (Figure 5i–l). The CV curves show the occurrence of the ORR during the cathodic scans, leading to an intense peak centered at ∼2.2 V vs Li^+^/Li. The subsequent anodic scan shows the currents associated to the OER, occurring through a first step at 3.5 V vs Li^+^/Li and a second one above 4.0 V vs Li^+^/Li. Among the investigated Li–O_2_ cells, the one based on 22BB displays the sharpest and most intense cathodic current peak (Figure 5a), indicating fast ORR kinetics. Instead, the cell based on 28BC (Figure 5b) exhibits the broadest and less intense cathodic current peak, suggesting slowest kinetics of the discharge reaction. The cells using 36BB (Figure 5c) and 39BB (Figure 5d) show an intermediate trend of the cathodic currents. On the other hand, the small differences observed for the OER peaks of the Li–O_2_ cells suggest a limited effect of the bare GDLs on the oxidation kinetics when insulating Li_2_O_2_ is massively formed during the ORR within the full potential range. The Nyquist plots after each CV cycle (Figure 5e–h) are fitted with the *R*_*e*_(*R*_1_*Q*_1_)(*R*_2_*Q*_2_)*Q*_*g*_ equivalent circuit (Table 3, and bottom-side scheme in Figure S4 in Supporting Information), instead those at the OCV are the same reported in the inset of Figure 3b,d,f,h, and Table 2 (see the top-side scheme in Figure S4 in Supporting Information). Compared to the one used to fit the Nyquist plots reported in Figure 3, an additional (*R*_2_*Q*_2_) element is included to discriminate the Li^+^ transfer and the SEI formation at the electrode/electrolyte interphase.^52^ The fitting of the Nyquist plots after the voltammetry cycle indicates interphase resistance (*R*_1_ + *R*_2_ in Table 3) of about 86 Ω for 22BB (Figure 5e), 115 Ω for 28BC (Figure 5f), 96 Ω for 36BB (Figure 5g), and 93 Ω for 39BB (Figure 5h). These low impedance values suggest a limited electrolyte decomposition during the ORR and OER, thus indicating the suitability of the GDLs for promoting efficient electrochemical reactions in the Li–O_2_ systems.^52^ Further proof of the efficiency of the electrochemical processes is given by the charge/discharge galvanostatic profiles of the Li–O_2_ cells recorded with no capacity limitation (Figure 5i–l). The cells using 22BB (Figure 5i), 28BC (Figure 5j), 36BB (Figure 5k), and 39BB (Figure 5l) achieve notable discharge areal capacities of 6.8, 7.4, 6.4, and 7.8 mA h cm^–2^, respectively, corresponding to cell capacities of 13.6, 14.8, 12.8, and 15.6 mA h, with a high Coulombic efficiency. It is worth noting that the different reversibility of CV tests in Figure 5a–d compared to the galvanostatic tests in Figure 5i–l may be attributed to the higher current values reached in the former compared to the latter. Thus, the galvanostatic test is performed at a constant current of 0.2 mA, while in the CV, the currents reach maximum values ranging from about 3 mA in discharge to about 1 mA in charge. Thus, the Li–O_2_ cell using 39BB as the cathodic support shows the best performance in terms of delivered capacity and Coulombic efficiency, indicating that the characteristics of this GDL, including low surface area and low porosity (see Table 1), are beneficial to attain the reversible Li + 1/2O_2_ ⇄ 1/2Li_2_O_2_ reaction.^53^

**Table 3:** NLLS Analyses of the Nyquist Plots Reported in Figure 5 Recorded by EIS after CV (Potential between 1.5 and 4.3 V vs Li^+^/Li) for the Li–O_2_ Cells Using the Investigated GDLs as Cathodes^a^

GDL	circuit	*R*_1_ [Ω]	*R*_2_ [Ω]	*R*_1_ + *R*_2_ [Ω]	χ^2^
22BB	*R*_e_(*R*_1_*Q*_1_)(*R*_2_*Q*_2_)*Q*_g_	7.4 ± 0.6	78.9 ± 0.8	86.3 ± 1.4	1 × 10^–4^
28BC	*R*_e_(*R*_1_*Q*_1_)(*R*_2_*Q*_2_)*Q*_g_	36.0 ± 1.4	78.7 ± 1.6	115 ± 3	7 × 10^–6^
36BB	*R*_e_(*R*_1_*Q*_1_)(*R*_2_*Q*_2_)*Q*_g_	17.8 ± 0.8	78.1 ± 0.9	95.9 ± 1.7	5 × 10^–5^
39BB	*R*_e_(*R*_1_*Q*_1_)(*R*_2_*Q*_2_)*Q*_g_	7.1 ± 0.9	86.1 ± 1.1	93.2 ± 2.0	1 × 10^–4^

a The NLLS fitting was performed with Boukamp software, and only χ^2^ values of the order of 10^–4^ or lower were accepted.^37,38^

**Figure 6:**
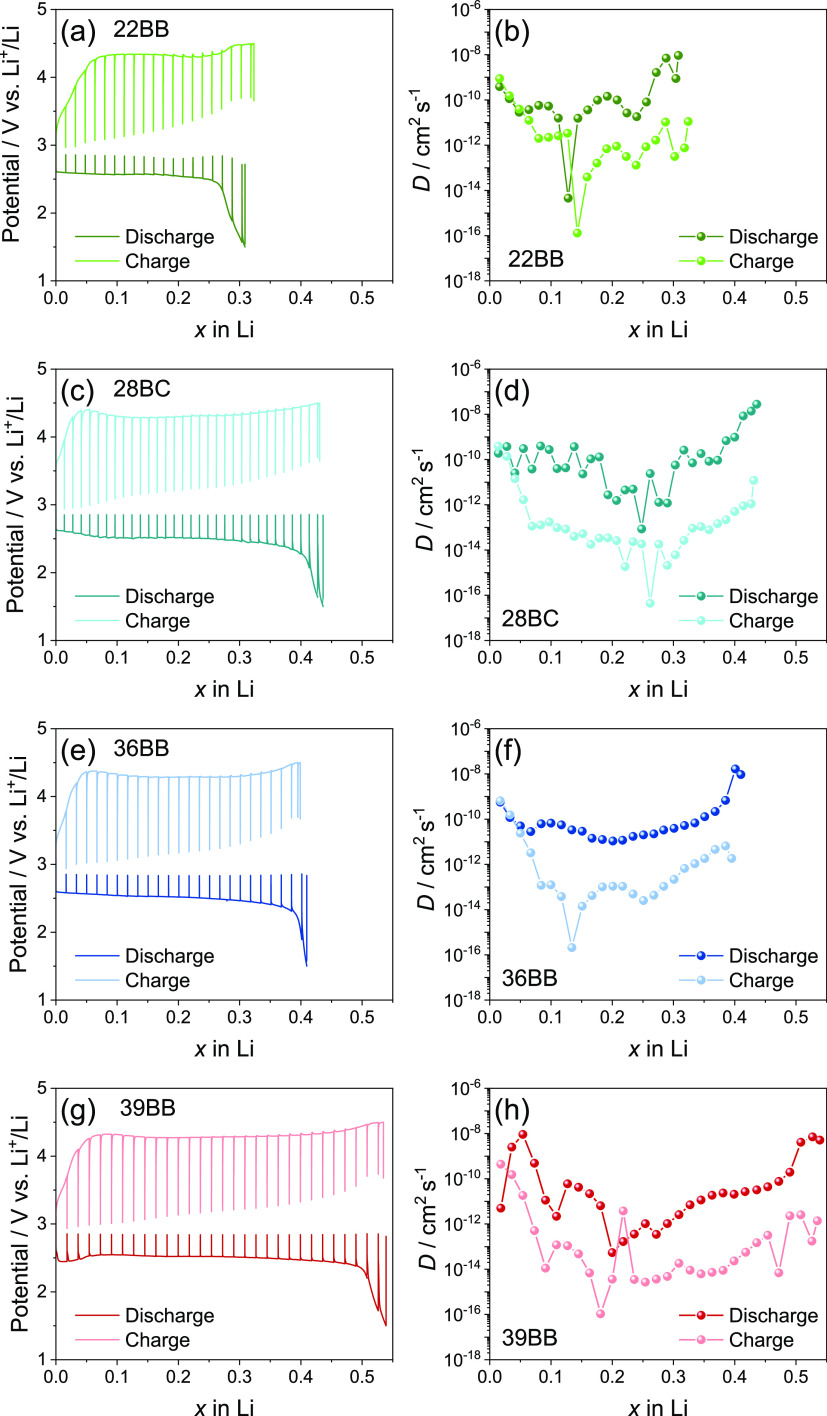
(a,c,e,g) GITT curves reporting the potential vs *x* and (b,d,f,h) *D* trends calculated by GITT equation (eq 1)^18,20,54^ at various SOCs for the Li–O_2_ cells using (a,b) 22BB, (c,d) 28BC, (e,f) 36BB, or (g,h) 39BB as cathodes (see maximum and minimum *D* values in in Table S1 in Supporting Information and the potential vs time GITT curves in Figure S7). Square current pulse: 0.4 mA; time of pulse: 1 h; potential relaxation step time: 1 h; and potential range: 1.5–4.5 V vs Li^+^/Li.

The electrochemical performances of the investigated GDLs in Li–O_2_ cells are further rationalized by determining the Li^+^ diffusion coefficient (*D*) at various SOCs using GITT (Figure 6).^54^ Typically, this technique evaluates the effect on *D* promoted by the exchange of a Li-equivalent fraction (*x*) within active materials designed for Li-ion batteries, such as Li_1–*x*_FePO_4_.^18,55^ More recent work reported the use of GITT for the evaluation of the diffusional features of Li–S batteries, considering the exchange of *x* in the Li_2*x*_S reaction products.^20^ In our case, Li–O_2_ cells represent three-phase (solid/liquid/gas) systems, which hinder the proper determination of the *x* value at the cathode side.^56^ Indeed, the exact mass of the electroactive specie on cathode, i.e., the oxygen on the GDL which is used only as the support for the electrochemical reaction, is practically complex to determine in particular in the cell setup used herein (i.e., CR2023 top-meshed coin cell in an excess of statical O_2_ gas). Therefore, we refer herein to the *x* equivalents exchanged within the Li metal anode, the mass of which can be easily determined, for the evaluation of the *D* values calculated through the GITT eq 1(54)
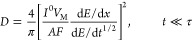
1where *I*^0^ (A) is the applied current, *V*_M_ is the Li molar volume (13.02 cm^3^ mol^–1^), *A* is the Li geometric area (1.54 cm^2^), *F* is the Faraday constant (96,485 C mol^–1^), τ is the diffusion time employed in the tests, d*E*/d*x* is obtained by derivation of the titration plots in Figure 6a,c,e,g, and d*E*/d*t*^1/2^ is determined by linear fitting of the relaxation potential vs *t*^1/2^ related to each current pulse (with *t* ≪ τ).^20^ Despite the above technique can help the rationalization of the Li–O_2_ battery behavior, the diffusion in the cell configuration adopted in this work avoids the actual deconvolution of the various factors, including Li^+^ and O_2_ transport, ORR/OER kinetics, nucleation and growth of Li_2_O_2_, and formation/decomposition of parasitic products, which are instead taken in whole by the “practical version” of the diffusion coefficient determined hereafter. Indeed, the complex nature of the battery hinders the full discerning of the various processes. In particular, the ion as well as the oxygen diffusion at the cathode/electrolyte interphase which may represent the rate-determining step of the cell, despite the contribution of the electrolyte and anode may be not completely excluded. Figure 6a,c,e,g shows the potential profiles recorded at quasi-equilibrium condition as a function of *x*, as achieved by the elaboration of the corresponding GITT potential vs time curves (Figure S7).^18,20^ Importantly, these data are consistent with the cell voltage profiles recorded during the galvanostatic charge/discharge cycling (see Figure 5) and reveal different *x* values for the various GDLs. Hence, maximum *x* values of 0.31, 0.44, 0.41, and 0.54 are observed during discharge for the cells using 22BB (Figure 6a), 28BC (Figure 6c), 36BB (Figure 6e), and 39BB (Figure 6g), respectively. These values indicate that 39BB is the most performant GDL for Li–O_2_ cells among the investigated ones. The trends of the *D* values achieved from GITT upon the change of *x* during the ORR/OER are reported in Figure 6b, d, f, and h for the cells using 22BB, 28BC, 36BB, and 39BB, respectively. For all the cells, the data show higher *D* values during discharge than during charge, thus accounting for a faster kinetics during the ORR than during the OER. This behavior is consistent with the differences of the reactants involved in the two processes, i.e., Li and O_2_ in the former while insulating Li_2_O_2_ in the latter.^14,57,58^ The data also reveal a decrease of *D* during the initial stages of the cell discharge and charge, where Li_2_O_2_ begins the deposition on the GDLs or it undergoes oxidation, respectively, due to the notable activation energy of the ORR and OER.^13^ Subsequently, *D* increases most likely due to the stabilization and consolidation of the electrode/electrolyte interphase, as already supported by EIS analyses (see Figure 5).

Table S1 in Supporting Information displays the maximum and minimum *D* calculated using GITT, indicating that 28BC leads to both the highest *D* value of 2.8 × 10^–8^ cm^2^ s^–1^ and the lowest one of 4.4 × 10^–17^ cm^2^ s^–1^. The other GDLs show intermediate *D*, ranging from 10^–8^ to 10^–16^ cm^2^ s^–1^, while the sample 39BB reveals the most suitable *D* values until the highest *x* of 0.55. Hence, GITT indicates the interplay between the GDL properties, including its surface characteristics, and the SOC of the Li–O_2_ cell in determining both the diffusional properties and the electrochemical performances. This behavior is associated with redox processes that involve multiple phases (i.e., solid, liquid, and gas) and formation of insulating species (Li_2_O_2_) and reaction intermediates including radicals and nucleophiles.^13^ Despite the complex response, the GITT analysis suggests the use of 39BB to ensure the most performant Li-equivalent exchange in Li–O_2_ cells, aiming at maximizing the discharge capacities of the latter. Indeed, previous work demonstrated that the growth of Li_2_O_2_ crystals follows a surface-mechanism in our cell setup.^14^ According to the above mechanism, the nucleation in the system leads to the formation of Li_2_O_2_ microparticles by direct-electrodeposition over the surface of the support, the size and distribution of which depend on the local current density. Hence, GDLs with lower porosity and surface, thus with the higher local current, can lead to the better performance due to the deposition of bigger Li_2_O_2_ micrometric particles distributed into the conductive framework, rather than small particles covering and possibly insulating the support.

With the aim of further understanding the nature of the *D* coefficient determined herein, we have performed polarization tests through galvanodynamic reduction scans on Li–Li and Li-GDL(39BB) cells in an O_2_ atmosphere. The data reported in Figure S8 in Supporting Information suggest a limiting current exceeding the value of 5 mA cm^–2^ for Li^+^ diffusion in the Li–Li symmetrical system and a complex trend for the Li-GDL(39BB) cell evolving with a double slope, suggesting a concomitant role of the O_2_ diffusion at lower currents in the Li–O_2_ cells.

### Use of the GDL Coated with MWCNTs**/**FLG in the Li–O_2_ Cell with Prolonged Cycling

**Figure 7:**
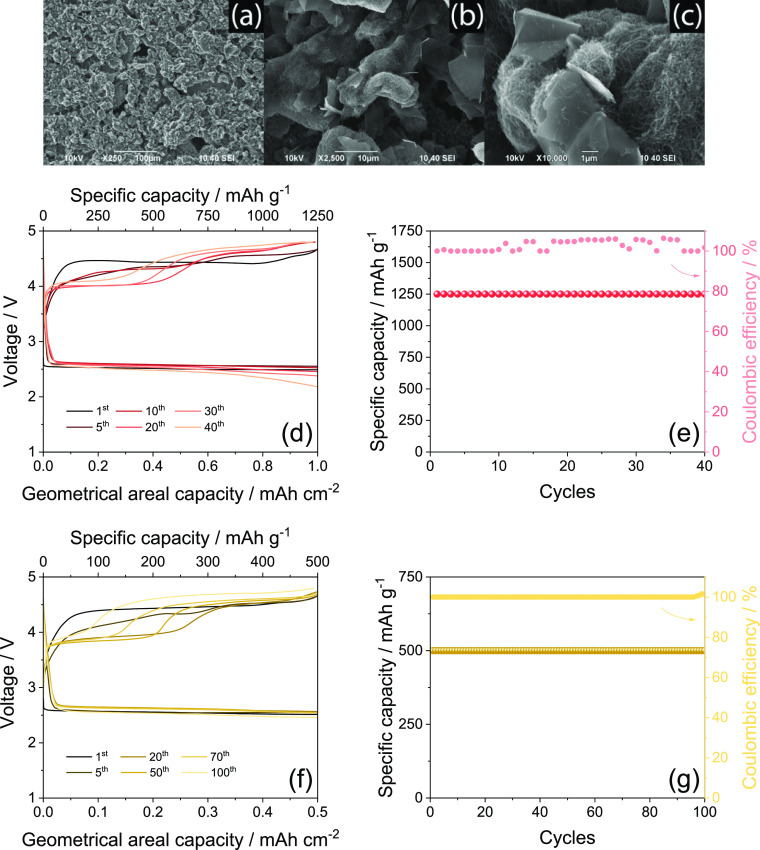
(a–c) SEM images at various magnifications of the electrode using the MWCNTs/FLG mixture coated onto the 39BB GDL (see the Experimental Section); (d,f) voltage profiles; and (e,g) corresponding specific capacity with Coulombic efficiency trends measured for Li–O_2_ cells using the 39BB GDL coated with MWCNTs/FLG as the cathode [MWCNTs/FLG loading of either (d,e) 0.8 mg cm^–2^ or (f,g) 1.0 mg cm^–2^ considering the GDL geometric area of 2.0 cm^2^]. The batteries are cycled at a constant current of 0.66 mA by limiting the capacity to either (d,e) 2 mA h (1 mA h cm^–2^) or (f,g) 1 mA h (0.5 mA h cm^–2^). In panels (d,f), bottom *x*-axes report the geometrical areal capacity (mA h cm^–2^), while top *x*-axes show the specific capacity (mA h g^–1^); in panels (e,g), right *y*-axis displays the Coulombic efficiency. Voltage range: 1.5–4.8 V.

According to the above GDL characterization, 39BB is subsequently selected as a suitable cathodic support for the realization of a practical Li–O_2_ battery based on a MWCNTs/FLG electrode. Figure 7 reports the SEM images at various magnifications of the electrode, alongside with the voltage profiles and corresponding specific capacity and Coulombic efficiency trends as a function of galvanostatic charge/discharge cycles of the corresponding Li–O_2_ cell. The SEM images show an electrode surface mainly formed by MWCNTs (Figure 7a) with a characteristic morphology including secondary particles with sizes ranging from 10 to 30 μm (Figure 7b) intimately curling up primary nanotubes.^14^ The SEM imaging also evidences the presence of FLG flakes, with sizes ranging from 1 to 10 μm and nanometric thickness, dispersed into the MWCNT framework (Figure 7b,c).^21^ The cell using the 39BB GDL coated with MWCNTs/FLG as the electrode is cycled at a constant current of 0.66 mA (geometrical areal value: 0.33 mA cm^–2^) by limiting the capacity to 2 mA h (geometrical areal value: 1 mA h cm^–2^) that corresponds to charge and discharge processes of 3 h each. The cell shows shapes of voltage profiles (Figure 7d) similar to those collected for the corresponding cell using the bare GDL (Figure 4d), although a remarkable three times higher current is reached upon the incorporation of MWCNTs and FLG. The cell reveals a Coulombic efficiency approaching 100%, which is actually achieved by the capacity limit, and a relevant specific capacity of 1250 mA h g^–1^ (as referred to the weight of the MWCNTs/FLG mixture) over 40 charge/discharge cycles (Figure 7e). Prospectively, a further increase of the cycle life of the cell may be achieved by tuning the MWCNTs/FLG weight ratio, as well as by activating the MWCNTs using thermal treatments under an N_2_ atmosphere as reported in our previous work.^14^ Literature papers suggest various additional strategies to limit the overvoltage and increase the cycle life of the Li–O_2_ cell.^14,36,59^ The first and simplest one consists on the decrease of the cell capacity limit to achieve the extended cycle life.^36^ We have adopted this strategy in Figure 7f,g by lowering the capacity limit from 2 mA h (geometrical areal value: 1 mA h cm^–2^) to 1 mA h (geometrical areal value: 0.5 mA h cm^–2^) in the Li–O_2_ cell using the 39BB coated with MWCNTs/FLG cycled at 0.66 mA. The new capacity limit, which corresponds to a gravimetric value of 500 mA h g^–1^, leads to the extension to the cell lifespan from 40 to 100 cycles, in agreement with literature work.^14^

Furthermore, the use of catalysts and redox mediators can actually lower the charge polarization and thus extend the cycle life due to the limited side reactions, such as the electrolyte degradation occurring in the Li–O_2_ cell.^27^ In addition, the use of a different electrolyte, such as ionic liquids, can change the reaction mechanism, lower the polarization, and extend the cycle life of the cell.^59^

**Figure 8:**
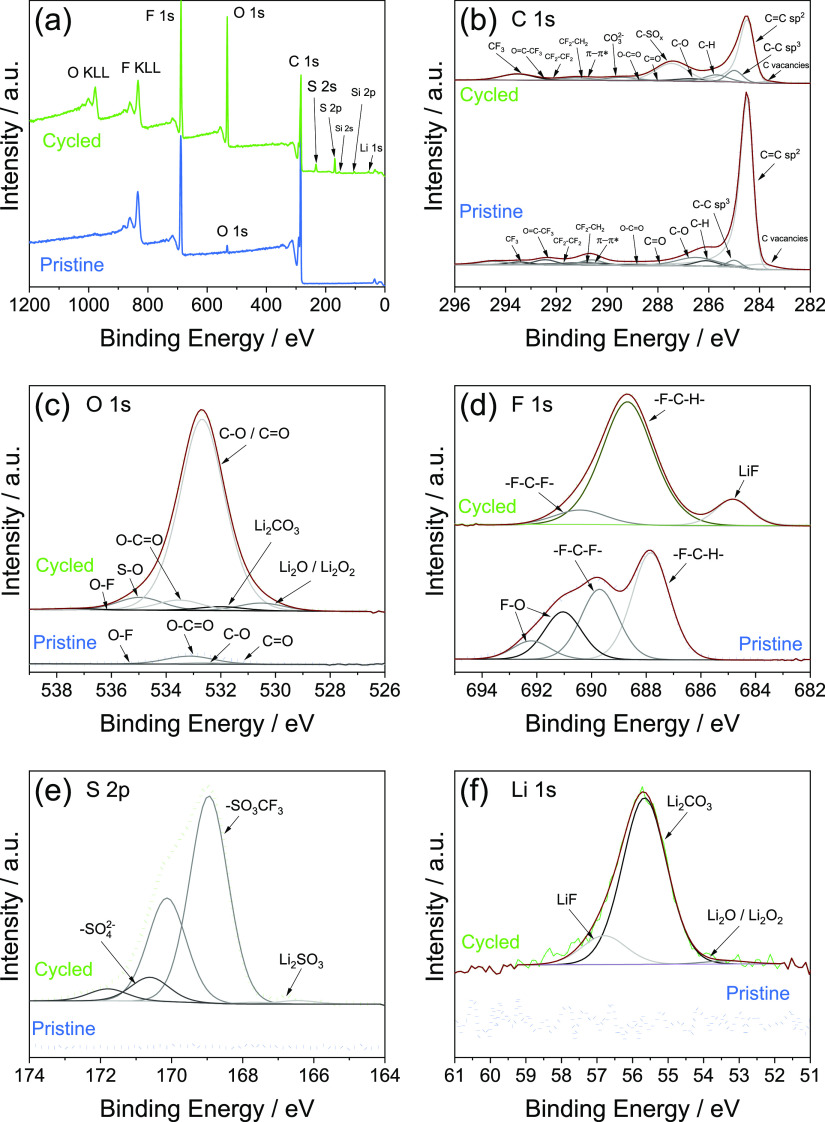
XPS measurements of the 39BB GDL coated with MWCNTs/FLG at the pristine state and after three cycles in Li–O_2_ cells at a constant current of 0.66 mA and capacity limited to 2 mA h (see the Experimental Section for details). In particular: (a) survey spectra and (b–f) high-resolution signals acquired in the (b) C 1s, (c) O 1s, (d) F 1s, (e) S 2p, and (f) Li 1s regions.

To examine the chemical composition of the SEI layer formed at the electrode/electrolyte interphase, a 39BB coated with the MWCNTs/FLG electrode is cycled in the Li–O_2_ cell with the same conditions of Figure 7d,e and subsequently retrieved from the cell. XPS measurements are performed on the cycled electrode and on a pristine one for comparison, and the results are reported in Figure 8. The survey spectra of pristine and cycled electrodes are reported in Figure 8a. The spectrum of the pristine electrode is dominated by the characteristic peaks related to C 1s and F 1s, likely related with the GDL substrate, FLG, and MWCNTs, and to the PVDF binder, respectively. A low amount of adsorbed oxygen at the sample surface is detected, possibly due to partial oxidation of one of the electrode components. After the third charge/discharge cycle, the survey spectrum of the electrode exhibits the expected C 1s, O 1s, and F 1s signals, along with additional peaks related to Li 1s and S 2p derived from the contact of the MWCNTs/FLG-coated 39BB electrode with the electrolyte solution. The presence of the Si peaks is originated from the glass fiber used as a separator in the cell. The relative atomic concentrations of C, O, F, S, and Li are quantified and reported in Table S2 in Supporting Information. Increase of O and F contents is observed at the surface of the cycled electrode compared to the pristine one, together with the decreased C atomic concentration. High-resolution C 1s, O 1s, F 1s, S 2p, and Li 1s XPS spectra are acquired and reported in Figure 8b–f. In the pristine electrode, the C 1s spectrum is deconvolved into seven peaks, ascribed to MWCNTs/FLG mixture compounds, at 283.7 ± 0.2, 284.5 ± 0.2, 285.0 ± 0.2, 286.5 ± 0.2, 287.9 ± 0.2, 288.9 ± 0.2, and 290.9 ± 0.2 eV (Figure 8b). They correspond to C vacancies, C=C (sp^2^-hybridized carbon), C–C (sp^3^-hybridized carbon), C–O (hydroxyl), C=O (carbonyl), O=C–O (carboxyl), and π–π* satellite peak, respectively.^60^ The presence of PVDF is associated with the appearance of five additional peaks centered at 286.1 ± 0.2 eV (attributed to the CH_2_ group), 290.6 ± 0.2 eV (CF_2_–CH_2_), 291.7 ± 0.2 eV (CF_2_–CF_2_), 292.4 ± 0.2 eV (O=C–CF_3_), and 293.5 ± 0.2 eV (CF_3_).^61^ The two components in the F 1s spectrum (Figure 8d), located at 687.8 ± 0.2 and 689.7 ± 0.2 eV, correspond to −F–C–H– and −F–C–F– groups, respectively, related to the PVDF binder.^62^ The additional components at higher binding energy (691.0 ± 0.2 and 692.2 ± 0.2 eV in Figure 8d) may be attributed to O bonded to a highly electronegative element such as F to form O–F bonds.^63,64^ Other authors^65,66^ suggested that the formation of the bump visible at > 692 eV caused by local charging effects of the PVDF binder during the analysis, related to the “negative charge trapping” within the PVDF. The O 1s spectrum (Figure 8c) can be deconvoluted into four peaks centered at 531.7 ± 0.2, 532.7 ± 0.2, 533.4, and 535.7 ± 0.2 eV, assigned to the C=O, C–O, O–C=O, and O–F groups.^67^ In the cycled electrode, the C 1s spectrum resembles to the one of the pristine electrode (Figure 8b). The most notable distinctions from the pristine sample include the appearance of two new components at 287.5 ± 0.2 and 290.0 ± 0.2 eV, identified as C–SO_*x*_ and CO_3_^2–^.^61,68^ The pronounced C–SO_*x*_ and the slightly noticeable CO_3_^2–^ signals, alongside with the increased −CF_3_ one in the C 1s spectrum, suggest the presence and possible decomposition of LiCF_3_SO_3_ conductive salt strongly adsorbed to the carbon electrode. The degradation of the salt with the formation of kinetically stable products at the SEI layer is confirmed by two distinct contributions in the O 1s spectrum (Figure 8c) at 532.0 ± 0.2 and 534.8 ± 0.2 eV attributed to CO_3_^2–^ in Li_2_CO_3_ and S–O groups, respectively.^65,69^ Additionally, the S 2p spectrum (Figure 8e) comprising the double split peaks at 168.9 ± 0.2 and 166.5 ± 0.2 eV validates the presence of −SO_3_CF_3_ and the formation of Li_2_SO_3_ as electrolyte degradation product.^70^ The additional peak at 170.6 eV is probably due to the chemisorption of oxygen, with the formation of SO_4_^2–^ species.^71^ The strong contribution of the Li_2_CO_3_ component at 55.7 ± 0.2 eV^72^ (532 ± 0.2 eV in the O 1s spectrum, Figure 8c) to the global Li 1s signal (Figure 8f) hinders the possibility of precisely evaluating the nature of the low intensity species at lower binding energy ∼53 ± 0.2 eV, precluding the distinction between Li_2_O and Li_2_O_2_ compounds. Although the expected LiF decomposition product of fluorinated salt during the discharge can dissolve during the charge,^65^ the F 1s spectrum (Figure 8d) reveals discernible LiF peak at 684.8 ± 0.2 eV (in the Li 1s spectrum at 56.8 ± 0.2 eV, Figure 8f), along with the components at 688.6 ± 0.2 eV and 690.4 ± 0.2 eV related to the PVDF binder.^73^ The slight shift toward higher binding energy of the latter two components compared to those for the pristine electrode can be ascribed to local charging effects.^66^

Overall, the XPS indicates that the SEI formed at the electrode surface in the Li–O_2_ cell under the setup adopted in this work is mainly formed by decomposition products of the LiCF_3_SO_3_ conducting salt and the TEGDME solvent (e.g., Li_2_SO_4_, LiF, and RCF_3_SO_3_), which are strongly adsorbed into a protective layer increasing the cycle life of the battery.

## Conclusions

Various GDLs indicated as 22BB, 28BC, 36BB, and 39BB have been characterized in terms of physical–chemical features, which were correlated to the performances of Li–O_2_ batteries using the GDLs as the cathode. The SEM-EDS analyses of the GDLs revealed different surface morphology and a composition based on carbon and PTFE binder. The XRD patterns of the GDLs indicated the presence of carbon with either graphitic or amorphous characters. The contents of the PTFE in the GDLs, determined through TGA, were found to be 17% for 22BB, 13% for both 28BC and 39BB, and 12% for 36BB. The BET analysis of N_2_ physisorption measurements indicated specific surface area of 39, 38, 31, and 13 m^2^ g^–1^ for 22BB, 28BC, 36BB, and 39BB, respectively, and total pore volumes between 0.10 and 0.14 cm^3^ g^–1^. The average pore diameter of the GDLs was found to be less than 3 nm. The electrochemical behavior of the GDLs as cathodic supports in Li–O_2_ cells was assessed through CV measurements performed in the potential range of 2.5–4.2 V vs Li^+^/Li, showing reversible ORR and OER occurring below 2.8 and above 3.6 V vs Li^+^/Li, respectively. After the first CV cycles, the currents associated to the ORR increased, suggesting an activation process associated to the stabilization of the electrode/electrolyte interphase and the formation of a suitable SEI at the electrode surface. On the other hand, the OER evidenced a more complex dependence between the CV profiles and the GDL nature due to the insulating character of the Li_2_O_2_ formed during the reaction in the absence of a specific catalyst. The EIS spectra recorded at OCV condition and after each CV cycles revealed initial resistances between 500 and 1500 Ω, which decreased to less than 100 Ω after CV, supporting the activation process that was particularly pronounced for 39BB (resistance after CV scan as low as 55 Ω). Galvanostatic charge/discharge cycling of the Li–O_2_ cells using the investigated GDLs were carried out by limiting the capacity to 2 mA h. The cells displayed promising performance, with reversible redox processes and a decrease of polarization after the first galvanostatic cycle. Additional CV tests using a wide potential range from 1.5 to 4.3 V vs Li^+^/Li showed resolved cathodic current peak, associated to the ORR and centered at 2.2 V vs Li^+^/Li. The ORR process was then reversed into a multi-step OER occurring at potentials between 3.5 and 4.3 V vs Li^+^/Li, with electrode/electrolyte interphase resistance limited to ∼100 Ω. The reversibility of the Li–O_2_ cells was further demonstrated by galvanostatic charge/discharge cycling without any capacity limitation, demonstrating geometrical areal capacities as high as 6.8, 7.4, 6.4, and 7.8 mA h cm^–2^ for cells using 22BB, 28BC, 36BB, and 39BB, respectively. Also, GITT measurements were performed to determine the practical Li^+^ diffusion coefficients (*D*) in the Li–O_2_ cells within the configuration adopted in this work using the various bare GDLs. The GITT data indicated that *D* is driven by both GDL properties and the SOC of the cell, with values in a vast range from 10^–8^ to 10^–17^ cm^2^ s^–1^. Importantly, the GITT analyses indicated that 39BB ensures the highest Li-equivalents (*x*) exchange, which, in turn, results in the highest cell discharge capacity among the investigated Li–O_2_ systems. In summary, the results reported in this work indicated that the less porous GDL (i.e., 39BB) represents the most suitable cathodic support for the realization of practical high-performance Li–O_2_ batteries. These characteristics have been attributed to the growth pathway of Li_2_O_2_ crystallites, which proceeds in our system according to the surface-mechanism over the sites of the carbon support. This direct-electrodeposition process forms bigger microparticles distributed into the conductive GDL in case of relatively high local current, low porosity and surface, instead smaller particles covering and possibly insulating the material in the case of the low local current, high porosity and surface. Accordingly, 39BB was coated with a MWCNTs/FLG mixture to further promote the electrochemical process, resulting in a Li–O_2_ battery with specific capacity as high as 1250 mA h g^–1^ (1 mA h cm^–2^) at ∼2.7 V discharge voltage with a high Coulombic efficiency over 40 cycles achieved at a current density of 0.33 mA cm^–2^ (specific current: 412.5 mA g^–1^). Further limitation of the capacity to 500 mA h g^–1^ (0.5 mA h cm^–2^) has led to the extension of the cell lifespan over 100 cycles. In addition, XPS on the cycled electrode suggested a cell stability promoted by the formation of a suitable SEI layer at the surface.

## Supporting Information Available

SEM images, DTG curves, and PSD measured for the investigated GDLs: 22BB, 28BC, 36BB, and 39BB; equivalent circuits used for the NLLS analyses; EIS Nyquist plots of Li–Li and GDL(39BB)-GDL(39BB) cells in an O_2_ atmosphere; polarization at the end of charge/discharge (overvoltage) as a function of the cycle number; GITT curves measured for the Li–O_2_ cells using the bare GDLs (22BB, 28BC, 36BB, or the 39BB) as cathodes; maximum and minimum *D* values calculated through GITT; galvanodynamic reduction scans Li–Li or Li-GDL(39BB) cell in an O_2_ atmosphere; and relative atomic element concentrations detected on pristine and cycled electrodes by XPS (PDF)

## References

[ref1] Larcher D.; Tarascon J.-M. Towards Greener and More Sustainable Batteries for Electrical Energy Storage. Nat. Chem. 2015, 7, 19–29. 10.1038/nchem.2085. 25515886

[ref2] Varzi A.; Thanner K.; Scipioni R.; Di Lecce D.; Hassoun J.; Dörfler S.; Altheus H.; Kaskel S.; Prehal C.; Freunberger S. A. Current Status and Future Perspectives of Lithium Metal Batteries. J. Power Sources 2020, 480, 228803 10.1016/j.jpowsour.2020.228803.

[ref3] Turcheniuk K.; Bondarev D.; Singhal V.; Yushin G. Ten Years Left to Redesign Lithium-Ion Batteries. Nature 2018, 559, 467–470. 10.1038/d41586-018-05752-3. 30046087

[ref4] Kwak W.-J.; Rosy; Sharon D.; Xia C.; Kim H.; Johnson L. R.; Bruce P. G.; Nazar L. F.; Sun Y.-K.; Frimer A. A.; Noked M.; Freunberger S. A.; Aurbach D. Lithium–Oxygen Batteries and Related Systems: Potential, Status, and Future. Chem. Rev. 2020, 120, 6626–6683. 10.1021/acs.chemrev.9b00609. 32134255

[ref5] Carbone L.; Greenbaum S. G.; Hassoun J. Lithium Sulfur and Lithium Oxygen Batteries: New Frontiers of Sustainable Energy Storage. Sustainable Energy Fuels 2017, 1, 228–247. 10.1039/C6SE00124F.

[ref6] Marangon V.; Hernandez-Rentero C.; Levchenko S.; Bianchini G.; Spagnolo D.; Caballero A.; Morales J.; Hassoun J. Lithium–Oxygen Battery Exploiting Highly Concentrated Glyme-Based Electrolytes. ACS Appl. Energy Mater. 2020, 3, 12263–12275. 10.1021/acsaem.0c02331.

[ref7] Van Noorden R. The Rechargeable Revolution: A Better Battery. Nature 2014, 507, 26–28. 10.1038/507026a. 24598624

[ref8] Abraham K. M. M. Prospects and Limits of Energy Storage in Batteries. J. Phys. Chem. Lett. 2015, 6, 830–844. 10.1021/jz5026273. 26262660

[ref9] Laoire C. O.; Mukerjee S.; Abraham K. M.; Plichta E. J.; Hendrickson M. A. Elucidating the Mechanism of Oxygen Reduction for Lithium-Air Battery Applications. J. Phys. Chem. C 2009, 113, 20127–20134. 10.1021/jp908090s.

[ref10] Di Lecce D.; Marangon V.; Jung H.-G.; Tominaga Y.; Greenbaum S.; Hassoun J. Glyme-Based Electrolytes: Suitable Solutions for next-Generation Lithium Batteries. Green Chem. 2022, 24, 1021–1048. 10.1039/D1GC03996B.

[ref11] Li Y.; Wang X.; Dong S.; Chen X.; Cui G. Recent Advances in Non-Aqueous Electrolyte for Rechargeable Li-O_2_ Batteries. Adv. Energy Mater. 2016, 6, 1600751 10.1002/aenm.201600751.

[ref12] Abraham K. M. Electrolyte-Directed Reactions of the Oxygen Electrode in Lithium-Air Batteries. J. Electrochem. Soc. 2014, 162, A3021–A3031. 10.1149/2.0041502jes.

[ref13] Hassoun J.; Croce F.; Armand M.; Scrosati B. Investigation of the O_2_ Electrochemistry in a Polymer Electrolyte Solid-State Cell. Angew. Chem., Int. Ed. 2011, 50, 2999–3002. 10.1002/anie.201006264. 21365721

[ref14] Carbone L.; Moro P. T.; Gobet M.; Munoz S.; Devany M.; Greenbaum S. G.; Hassoun J. Enhanced Lithium Oxygen Battery Using a Glyme Electrolyte and Carbon Nanotubes. ACS Appl. Mater. Interfaces 2018, 10, 16367–16375. 10.1021/acsami.7b19544. 29676560

[ref15] Elia G. A.; Park J.-B.; Sun Y.-K.; Scrosati B.; Hassoun J. Role of the Lithium Salt in the Performance of Lithium- Oxygen Batteries: A Comparative Study. ChemElectroChem 2014, 1, 47–50. 10.1002/celc.201300160.

[ref16] Tang K.; Yu X.; Sun J.; Li H.; Huang X. Kinetic Analysis on LiFePO_4_ Thin Films by CV, GITT, and EIS. Electrochim. Acta 2011, 56, 4869–4875. 10.1016/j.electacta.2011.02.119.

[ref17] Molenda J.; Ojczyk W.; Świerczek K.; Zając W.; Krok F.; Dygas J.; Liu R.-S. Diffusional Mechanism of Deintercalation in LiFe_1–y_Mn_y_PO_4_ Cathode Material. Solid State Ionics 2006, 177, 2617–2624. 10.1016/j.ssi.2006.03.047.

[ref18] Di Lecce D.; Hassoun J. Lithium Transport Properties in LiMn_1α_Fe_α_PO_4_ Olivine Cathodes. J. Phys. Chem. C 2015, 119, 20855–20863. 10.1021/acs.jpcc.5b06727.

[ref19] Brutti S.; Manzi J.; Meggiolaro D.; Vitucci F. M.; Trequattrini F.; Paolone A.; Palumbo O. Interplay between Local Structure and Transport Properties in Iron-Doped LiCoPO_4_ Olivines. J. Mater. Chem. A 2017, 5, 14020–14030. 10.1039/C7TA03161K.

[ref20] Lama F. L.; Marangon V.; Caballero Á.; Morales J.; Hassoun J. Diffusional Features of a Lithium-Sulfur Battery Exploiting Highly Microporous Activated Carbon. ChemSusChem 2023, 16, e202202095 10.1002/cssc.202202095. 36562306

[ref21] Marangon V.; Barcaro E.; Minnetti L.; Brehm W.; Bonaccorso F.; Pellegrini V.; Hassoun J. Current Collectors Based on Multiwalled Carbon-Nanotubes and Few-Layer Graphene for Enhancing the Conversion Process in Scalable Lithium-Sulfur Battery. Nano Res. 2023, 16, 8433–8447. 10.1007/s12274-022-5364-5.

[ref22] Lu Y.-C.; Shao-Horn Y. Probing the Reaction Kinetics of the Charge Reactions of Nonaqueous Li–O_2_ Batteries. J. Phys. Chem. Lett. 2013, 4, 93–99. 10.1021/jz3018368. 26291218

[ref23] Younesi R.; Hahlin M.; Edström K. Surface Characterization of the Carbon Cathode and the Lithium Anode of Li–O_2_ Batteries Using LiClO_4_ or LiBOB Salts. ACS Appl. Mater. Interfaces 2013, 5, 1333–1341. 10.1021/am3026129. 23336349

[ref24] Ottakam Thotiyl M. M.; Freunberger S. A.; Peng Z.; Bruce P. G. The Carbon Electrode in Nonaqueous Li-O_2_ Cells. J. Am. Chem. Soc. 2013, 135, 494–500. 10.1021/ja310258x. 23190204

[ref25] Li F.; Tang D. M.; Chen Y.; Golberg D.; Kitaura H.; Zhang T.; Yamada A.; Zhou H. Ru/ITO: A Carbon-Free Cathode for Nonaqueous Li-O_2_ Battery. Nano Lett. 2013, 13, 4702–4707. 10.1021/nl402213h. 24063602

[ref26] Gittleson F. S.; Ryu W.-H.; Schwab M.; Tong X.; Taylor A. D. Pt and Pd Catalyzed Oxidation of Li_2_O_2_ and DMSO during Li-O_2_ Battery Charging. Chem. Commun. 2016, 52, 6605–6608. 10.1039/C6CC01778A. 27111589

[ref27] Lu Y. C.; Xu Z.; Gasteiger H. A.; Chen S.; Hamad-Schifferli K.; Shao-Horn Y. Platinum-Gold Nanoparticles: A Highly Active Bifunctional Electrocatalyst for Rechargeable Lithium-Air Batteries. J. Am. Chem. Soc. 2010, 132, 12170–12171. 10.1021/ja1036572. 20527774

[ref28] Jeong Y. S.; Park J.-B.; Jung H.-G.; Kim J.; Luo X.; Lu J.; Curtiss L.; Amine K.; Sun Y.-K.; Scrosati B.; Lee Y. J. Study on the Catalytic Activity of Noble Metal Nanoparticles on Reduced Graphene Oxide for Oxygen Evolution Reactions in Lithium-Air Batteries. Nano Lett. 2015, 15, 4261–4268. 10.1021/nl504425h. 26115340

[ref29] Liu Z.; Zhao Z.; Zhang W.; Huang Y.; Liu Y.; Wu D.; Wang L.; Chou S. Toward high-performance Lithium-oxygen Batteries with Cobalt-based Transition Metal Oxide Catalysts: Advanced Strategies and Mechanical Insights. InfoMat 2022, 4, e12260 10.1002/inf2.12260.

[ref30] Chen L. Y.; Guo X. W.; Han J. H.; Liu P.; Xu X. D.; Hirata A.; Chen M. W. Nanoporous Metal/Oxide Hybrid Materials for Rechargeable Lithium–Oxygen Batteries. J. Mater. Chem. A 2015, 3, 3620–3626. 10.1039/C4TA05738D.

[ref31] Yin J.; Carlin J. M.; Kim J.; Li Z.; Park J. H.; Patel B.; Chakrapani S.; Lee S.; Joo Y. L. Synergy Between Metal Oxide Nanofibers and Graphene Nanoribbons for Rechargeable Lithium-Oxygen Battery Cathodes. Adv. Energy Mater. 2015, 5, 1401412 10.1002/aenm.201401412.

[ref32] Liu Q.-C.; Xu J.-J.; Xu D.; Zhang X.-B. Flexible Lithium–Oxygen Battery Based on a Recoverable Cathode. Nat. Commun. 2015, 6, 7892 10.1038/ncomms8892. 26235205 PMC4532833

[ref33] Del Rio Castillo A. E.; Pellegrini V.; Ansaldo A.; Ricciardella F.; Sun H.; Marasco L.; Buha J.; Dang Z.; Gagliani L.; Lago E.; Curreli N.; Gentiluomo S.; Palazon F.; Prato M.; Oropesa-Nuñez R.; Toth P. S.; Mantero E.; Crugliano M.; Gamucci A.; Tomadin A.; Polini M.; Bonaccorso F. High-Yield Production of 2D Crystals by Wet-Jet Milling. Mater. Horiz. 2018, 5, 890–904. 10.1039/C8MH00487K.

[ref34] Brunauer S.; Emmett P. H.; Teller E. Adsorption of Gases in Multimolecular Layers. J. Am. Chem. Soc. 1938, 60, 309–319. 10.1021/ja01269a023.

[ref35] Ravikovitch P. I.; Vishnyakov A.; Neimark A. v. Density Functional Theories and Molecular Simulations of Adsorption and Phase Transitions in Nanopores. Phys. Rev. E 2001, 64, 011602 10.1103/PhysRevE.64.011602. 11461265

[ref36] Jung H. G.; Hassoun J.; Park J. B.; Sun Y. K.; Scrosati B. An Improved High-Performance Lithium-Air Battery. Nat. Chem. 2012, 4, 579–585. 10.1038/nchem.1376. 22717445

[ref37] Boukamp B. A Nonlinear Least Squares Fit procedure for analysis of immittance data of electrochemical systems. Solid State Ionics 1986, 20, 31–44. 10.1016/0167-2738(86)90031-7.

[ref38] Boukamp B. A Package for Impedance/Admittance Data Analysis. Solid State Ionics 1986, 18–19, 136–140. 10.1016/0167-2738(86)90100-1.

[ref39] Arrebola J. C.; Caballero A.; Hernán L.; Morales J. Graphitized Carbons of Variable Morphology and Crystallinity: A Comparative Study of Their Performance in Lithium Cells. J. Electrochem. Soc. 2009, 156, A986 10.1149/1.3231489.

[ref40] Ruland W.; Smarsly B. X-Ray Scattering of Non-Graphitic Carbon: An Improved Method of Evaluation. J. Appl. Crystallogr. 2002, 35, 624–633. 10.1107/S0021889802011007.

[ref41] Dhillo R. K.; Singh S.; Kumar R. 150MeV Nickel Ion Beam Irradiation Effects on Polytetrafluoroethylene (PTFE) Polymer. Nucl. Instrum. Methods Phys. Res., Sect. B 2010, 268, 2189–2192. 10.1016/j.nimb.2010.02.085.

[ref42] Nasef M. M. Thermal Stability of Radiation Grafted PTFE-g-Polystyrene Sulfonic Acid Membranes. Polym. Degrad. Stab. 2000, 68, 231–238. 10.1016/S0141-3910(00)00005-7.

[ref43] Thommes M.; Kaneko K.; Neimark A. V.; Olivier J. P.; Rodriguez-Reinoso F.; Rouquerol J.; Sing K. S. W. Physisorption of Gases, with Special Reference to the Evaluation of Surface Area and Pore Size Distribution (IUPAC Technical Report). Pure Appl. Chem. 2015, 87, 1051–1069. 10.1515/pac-2014-1117.

[ref44] Nomura A.; Ito K.; Kubo Y. CNT Sheet Air Electrode for the Development of Ultra-High Cell Capacity in Lithium-Air Batteries. Sci. Rep. 2017, 7, 45596 10.1038/srep45596. 28378746 PMC5381228

[ref45] Zeng J.; Nair J. R.; Francia C.; Bodoardo S.; Penazzi N. Aprotic Li–O_2_ Cells: Gas Diffusion Layer (GDL) as Catalyst Free Cathode and Tetraglyme/LiClO_4_ as Electrolyte. Solid State Ionics 2014, 262, 160–164. 10.1016/j.ssi.2013.09.032.

[ref46] Aurbach D. Review of Selected Electrode–Solution Interactions Which Determine the Performance of Li and Li Ion Batteries. J. Power Sources 2000, 89, 206–218. 10.1016/S0378-7753(00)00431-6.

[ref47] Jung H.-G.; Kim H.-S.; Park J.-B.; Oh I.-H.; Hassoun J.; Yoon C. S.; Scrosati B.; Sun Y.-K. A Transmission Electron Microscopy Study of the Electrochemical Process of Lithium-Oxygen Cells. Nano Lett. 2012, 12, 4333–4335. 10.1021/nl302066d. 22812655

[ref48] Horwitz G.; Calvo E. J.; Méndez De Leo L. P.; de la Llave E. Electrochemical Stability of Glyme-Based Electrolytes for Li–O_2_ Batteries Studied by in Situ Infrared Spectroscopy. Phys. Chem. Chem. Phys. 2020, 22, 16615–16623. 10.1039/D0CP02568B. 32671355

[ref49] Black R.; Oh S. H.; Lee J.-H.; Yim T.; Adams B.; Nazar L. F. Screening for Superoxide Reactivity in Li-O_2_ Batteries: Effect on Li_2_O_2_ /LiOH Crystallization. J. Am. Chem. Soc. 2012, 134, 2902–2905. 10.1021/ja2111543. 22283803

[ref50] Gallant B. M.; Kwabi D. G.; Mitchell R. R.; Zhou J.; Thompson C. V.; Shao-Horn Y. Influence of Li_2_O_2_ Morphology on Oxygen Reduction and Evolution Kinetics in Li–O_2_ Batteries. Energy Environ. Sci. 2013, 6, 2518 10.1039/c3ee40998h.

[ref51] Jing Y.; Zhou Z. Computational Insights into Oxygen Reduction Reaction and Initial Li_2_O_2_ Nucleation on Pristine and N-Doped Graphene in Li-O_2_ Batteries. ACS Catal. 2015, 5, 4309–4317. 10.1021/acscatal.5b00332.

[ref52] Elia G. A.; Bresser D.; Reiter J.; Oberhumer P.; Sun Y.-K.; Scrosati B.; Passerini S.; Hassoun J. Interphase Evolution of a Lithium-Ion/Oxygen Battery. ACS Appl. Mater. Interfaces 2015, 7, 22638–22643. 10.1021/acsami.5b07414. 26389522

[ref53] Lu Y. C.; Gasteiger H. A.; Shao-Horn Y. Catalytic Activity Trends of Oxygen Reduction Reaction for Nonaqueous Li-Air Batteries. J. Am. Chem. Soc. 2011, 133, 19048–19051. 10.1021/ja208608s. 22044022

[ref54] Weppner W.; Huggins R. A. Determination of the Kinetic Parameters of Mixed-Conducting Electrodes and Application to the System Li_3_Sb. J. Electrochem. Soc. 1977, 124, 1569–1578. 10.1149/1.2133112.

[ref55] Tealdi C.; Heath J.; Islam M. S. Feeling the Strain: Enhancing Ionic Transport in Olivine Phosphate Cathodes for Li- and Na-Ion Batteries through Strain Effects. J. Mater. Chem. A 2016, 4, 6998–7004. 10.1039/C5TA09418F.

[ref56] Ryu W. H.; Gittleson F. S.; Schwab M.; Goh T.; Taylor A. D. A Mesoporous Catalytic Membrane Architecture for Lithium-Oxygen Battery Systems. Nano Lett. 2015, 15, 434–441. 10.1021/nl503760n. 25546408

[ref57] Zhang L.; Xia Z. Mechanisms of Oxygen Reduction Reaction on Nitrogen-Doped Graphene for Fuel Cells. J. Phys. Chem. C 2011, 115, 11170–11176. 10.1021/jp201991j.

[ref58] Mahne N.; Fontaine O.; Thotiyl M. O.; Wilkening M.; Freunberger S. A. Mechanism and Performance of Lithium-Oxygen Batteries-a Perspective. Chem. Sci. 2017, 8, 6716–6729. 10.1039/c7sc02519j. 29147497 PMC5643885

[ref59] Elia G. A.; Hassoun J.; Kwak W.-J.; Sun Y.-K.; Scrosati B.; Mueller F.; Bresser D.; Passerini S.; Oberhumer P.; Tsiouvaras N.; Reiter J. An Advanced Lithium–Air Battery Exploiting an Ionic Liquid-Based Electrolyte. Nano Lett. 2014, 14, 6572–6577. 10.1021/nl5031985. 25329836

[ref60] Morgan D. J. Comments on the XPS Analysis of Carbon Materials. C 2021, 7, 51 10.3390/c7030051.

[ref61] Bodenes L.; Dedryvère R.; Martinez H.; Fischer F.; Tessier C.; Pérès J.-P. Lithium-Ion Batteries Working at 85°C: Aging Phenomena and Electrode/Electrolyte Interfaces Studied by XPS. J. Electrochem. Soc. 2012, 159, A1739–A1746. 10.1149/2.061210jes.

[ref62] Viswanath P.; Yoshimura M. Light-Induced Reversible Phase Transition in Polyvinylidene Fluoride-Based Nanocomposites. SN Appl. Sci. 2019, 1, 1519 10.1007/s42452-019-1564-3.

[ref63] Bartnik A.; Lisowski W.; Sobczak J.; Wachulak P.; Budner B.; Korczyc B.; Fiedorowicz H. Simultaneous Treatment of Polymer Surface by EUV Radiation and Ionized Nitrogen. Appl. Phys. A: Solids Surf. 2012, 109, 39–43. 10.1007/s00339-012-7243-5.

[ref64] Wijaya O.; Hartmann P.; Younesi R.; Markovits I. I. E.; Rinaldi A.; Janek J.; Yazami R. A Gamma Fluorinated Ether as an Additive for Enhanced Oxygen Activity in Li–O_2_ Batteries. J. Mater. Chem. A 2015, 3, 19061–19067. 10.1039/C5TA03439F.

[ref65] Guéguen A.; Novák P.; Berg E. J. XPS Study of the Interface Evolution of Carbonaceous Electrodes for Li-O_2_ Batteries during the 1st Cycle. J. Electrochem. Soc. 2016, 163, A2545–A2550. 10.1149/2.0351613jes.

[ref66] Leanza D.; Vaz C. A. F.; Novák P.; El Kazzi M. Instability of PVDF Binder in the LiFePO_4_*versus* Li_4_Ti_5_O_12_ Li-Ion Battery Cell. Helv. Chim. Acta 2021, 104, e2000183 10.1002/hlca.202000183.

[ref67] Kaspar P.; Sobola D.; Částková K.; Dallaev R.; ŠT’astná E.; Sedlák P.; Knápek A.; Trčka T.; Holcman V. Case Study of Polyvinylidene Fluoride Doping by Carbon Nanotubes. Materials 2021, 14, 1428 10.3390/ma14061428. 33804184 PMC8001382

[ref68] Wang Q.; Yao Z.; Zhao C.; Verhallen T.; Tabor D. P.; Liu M.; Ooms F.; Kang F.; Aspuru-Guzik A.; Hu Y.-S.; Wagemaker M.; Li B. Interface Chemistry of an Amide Electrolyte for Highly Reversible Lithium Metal Batteries. Nat. Commun. 2020, 11, 4188 10.1038/s41467-020-17976-x. 32826904 PMC7442789

[ref69] Parry V.; Berthomé G.; Joud J.-C.; Lemaire O.; Franco A. A. XPS Investigations of the Proton Exchange Membrane Fuel Cell Active Layers Aging: Characterization of the Mitigating Role of an Anodic CO Contamination on Cathode Degradation. J. Power Sources 2011, 196, 2530–2538. 10.1016/j.jpowsour.2010.11.027.

[ref70] Agostini M.; Xiong S.; Matic A.; Hassoun J. Polysulfide-Containing Glyme-Based Electrolytes for Lithium Sulfur Battery. Chem. Mater. 2015, 27, 4604–4611. 10.1021/acs.chemmater.5b00896.

[ref71] Rotte N. K.; Naresh V.; Muduli S.; Reddy V.; Srikanth V. V. S.; Martha S. K. Microwave Aided Scalable Synthesis of Sulfur, Nitrogen Co-Doped Few-Layered Graphene Material for High-Performance Supercapacitors. Electrochim. Acta 2020, 363, 137209 10.1016/j.electacta.2020.137209.

[ref72] Wood K. N.; Teeter G. XPS on Li-Battery-Related Compounds: Analysis of Inorganic SEI Phases and a Methodology for Charge Correction. ACS Appl. Energy Mater. 2018, 1, 4493–4504. 10.1021/acsaem.8b00406.

[ref73] Nandasiri M. I.; Camacho-Forero L. E.; Schwarz A. M.; Shutthanandan V.; Thevuthasan S.; Balbuena P. B.; Mueller K. T.; Murugesan V. In Situ Chemical Imaging of Solid-Electrolyte Interphase Layer Evolution in Li–S Batteries. Chem. Mater. 2017, 29, 4728–4737. 10.1021/acs.chemmater.7b00374.

