# Supplementary material for: Influence of Ion Diffusion on the Lithium–Oxygen Electrochemical Process and Battery Application Using Carbon Nanotubes–Graphene Substrate
Source: ACS Appl Mater Interfaces. 2023 Aug 8;15(33):39218–33. doi: 10.1021/acsami.3c05240 (PMC10450645; doi:10.1021/acsami.3c05240)
Supplement: Supplementary file 1 — am3c05240_si_001.pdf [file am3c05240_si_001.pdf]

**Supporting Information**

# Influence of Ion Diffusion on the Lithium-Oxygen Electrochemical Process and Battery Application using Carbon Nanotubes-Graphene Substrate

Stanislav Levchenko[a,#], Vittorio Marangon[a,b,#], Sebastiano Bellani[c], Lea Pasquale[d] Francesco Bonaccorso[b,c], Vittorio Pellegrini[b,c], and Jusef Hassoun[a,b,e,*]

[a] *Department of Chemical, Pharmaceutical and Agricultural Sciences, University of Ferrara, Via Fossato di Mortara 17, Ferrara, 44121, Italy*

[b] *Graphene Labs, Istituto Italiano di Tecnologia, via Morego 30, Genoa, 16163, Italy*

[c] *BeDimensional S.p.A., Lungotorrente Secca 30R, 16163 Genoa, Italy*

[d] *Materials Characterization Facility, Istituto Italiano di Tecnologia, via Morego 30, Genova, 16163 Italy*

[e] *National Interuniversity Consortium of Materials Science and Technology (INSTM), University of Ferrara Research Unit, Via Fossato di Mortara, 17, 44121, Ferrara, Italy*

[#] Authors equally contributed

*Corresponding author. E-mail addresses: jusef.hassoun@unife.it, jusef.hassoun@iit.it.

Pages number: 11

Figures number: 8

Tables number: 2

Additional SEM images on the surface of the **(a)** 22BB, **(b)** 28BC, **(c)** 36BB and **(d)** 39BB GDLs recorded in secondary electrons mode, with higher magnification compared to the ones reported in Figure 1 of the Manuscript.

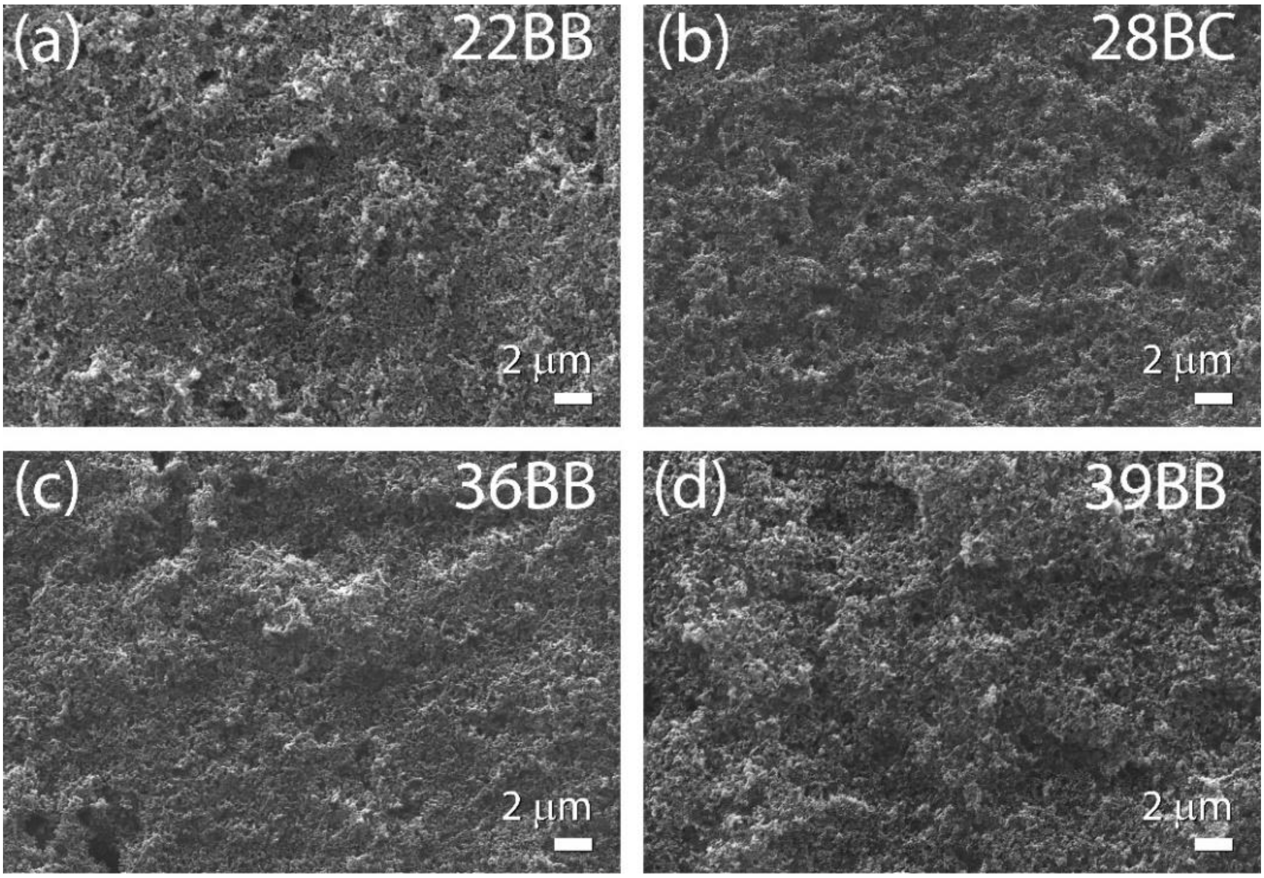


**Figure S1.** SEM images recorded on the surface of the **(a)** 22BB, **(b)** 28BC, **(c)** 36BB and **(d)** 39BB GDLs in secondary electrons mode.

Figure S2 shows the DTG curves measured for the investigated GDLs, *i.e.*, 22BB, 28BC, 36BB and 39BB, as derived from the TGA data reported in Figure 2 of the Manuscript. For all the GDLs, the main weight losses take place between 500 and 550 °C and are ascribed to the decomposition of the PTFE binder.[1] In particular, 22BB exhibits the highest binder content, and additional weight losses in the 50 – 400 °C temperature range which may suggest relevant absorption ability of moisture and solvents promoted by a more extended surface area of this GDL compared to the others.

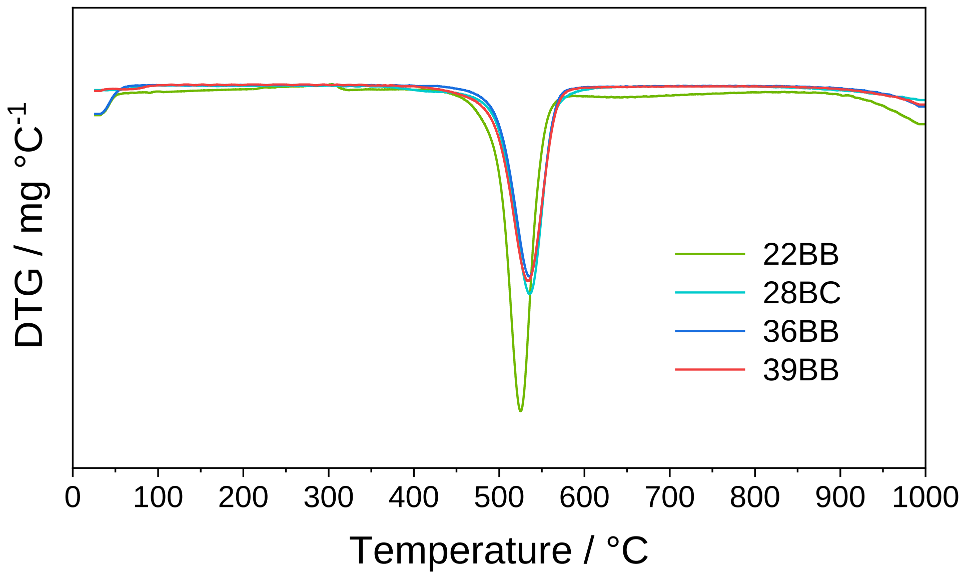


**Figure S2.** DTG curves derived from the TGA data measured for the 22BB, 28BC, 36BB and 39BB displayed in Figure 2 in the Manuscript.

Figure S3 displays the pore size distributions and cumulative pore volumes estimated for 22BB (Figure S3a), 28BC (Figure S3b), 36BB (Figure S3c) and 39BB (Figure S3d) GDL samples using $N_2$ adsorption data (Figure 2 in the Manuscript) after application of NLDFT method. The outcomes show for the GDLs pores diameter near 3 nm, and porosity gradually decreasing from 22BB, to 28BC, 36BB and 39BB.

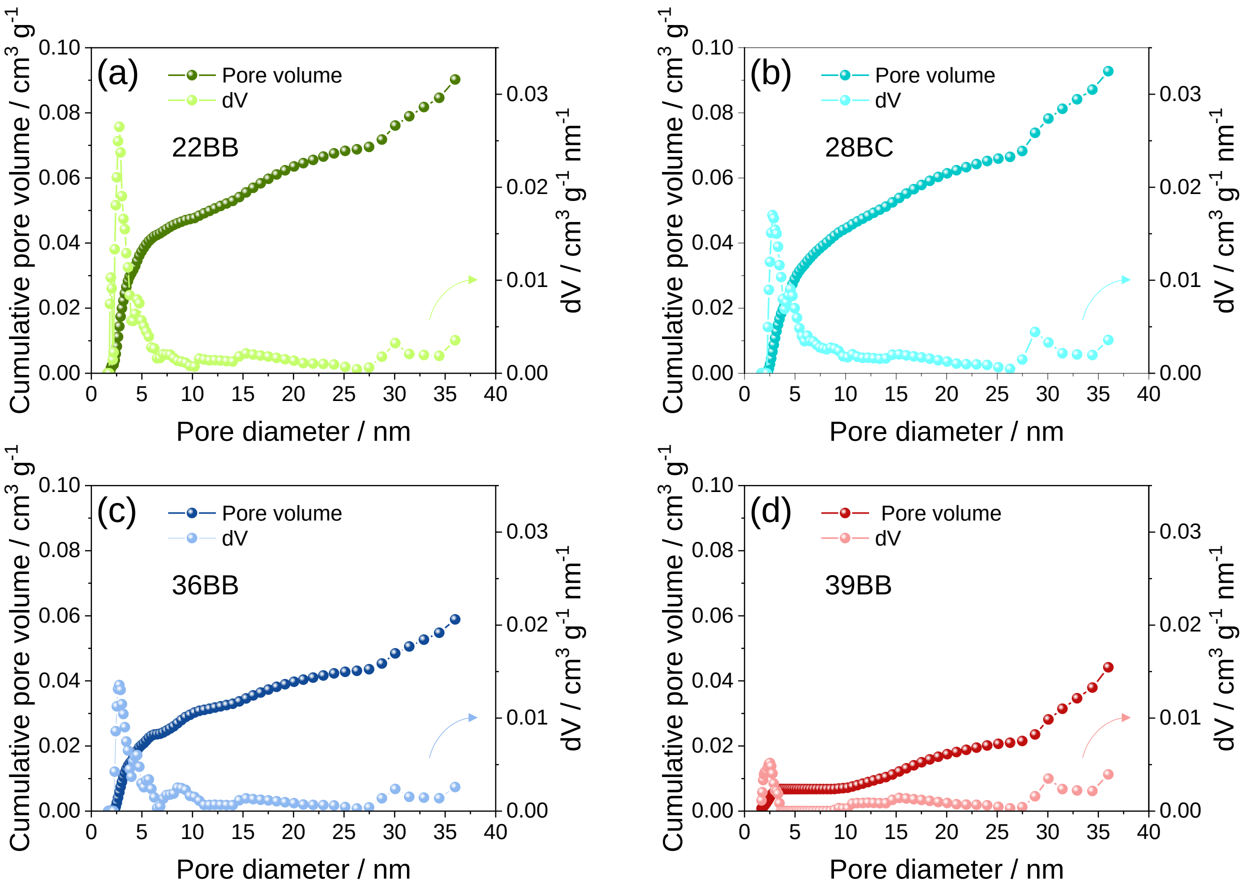


**Figure S3.** Pore size distribution and cumulative pore volume calculated for **(a)** 22BB, **(b)** 28BC, **(c)** 36BB and **(d)** 39BB using NLDFT method applied to the $N_2$ adsorption data (see Figure 2 of the Manuscript).

Figure S4 reports in the top and bottom side, respectively, a schematic diagram of the equivalent circuit used for NLLS analysis of the SEI Nyquist plots showed in Figure 3 (b, d, f, h) and Figure 5 (e-h) in the Manuscript.

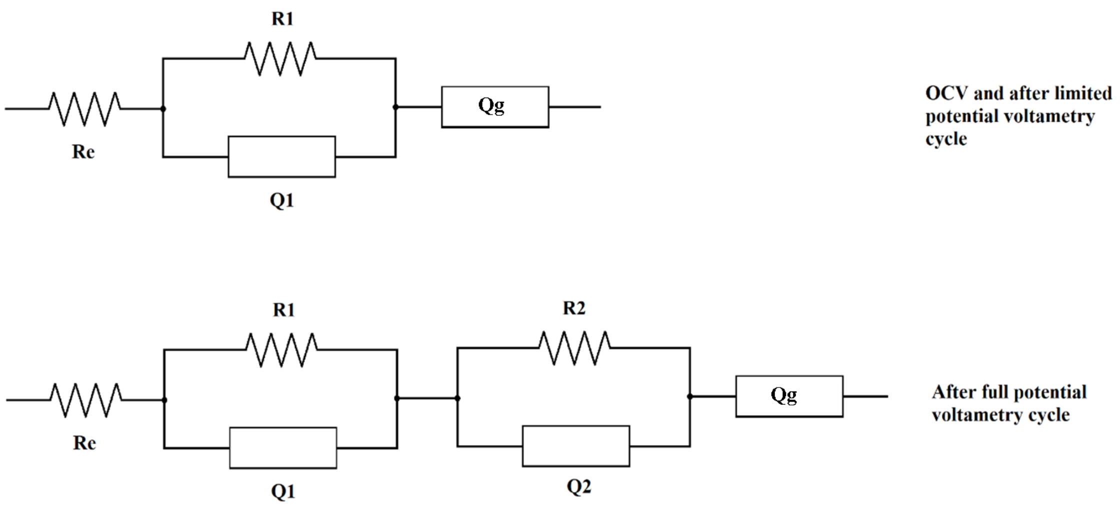


**Figure S4.** Equivalent circuits used for the non-linear least-squares (NLLS) analyses performed on the Nyquist plots reported in **(top-side)** Figure 3 (b, d, f, h) and **(bottom-side)** Figure 5 (e-h), recorded by EIS on GDL half-cell upon CV.

Figure S5 reports the EIS measurements are carried out on symmetric Li-Li cell (panel a) and GDL(39BB)-GDL(39BB) cell (panel b) in $O_2$ atmosphere at the OCV. The Figure S6a shows for the symmetric Li-Li cell a Nyquist plot including a semicircle at medium-high frequency ascribed to the electrode/electrolyte interphase, and a low frequency contribute related with the semi-finite Warburg-type Li+ diffusion, with a resistance around 100 Ω. Figure S6b indicates for the GDL(39BB)/GDL(39BB) cell a wide and noisy semicircle likely ascribed to possible side reaction of the electrolyte or ion diffusion, with a very large resistance value, i.e., extending 10000 Ω due to the absence of Li-source in the electrodes.

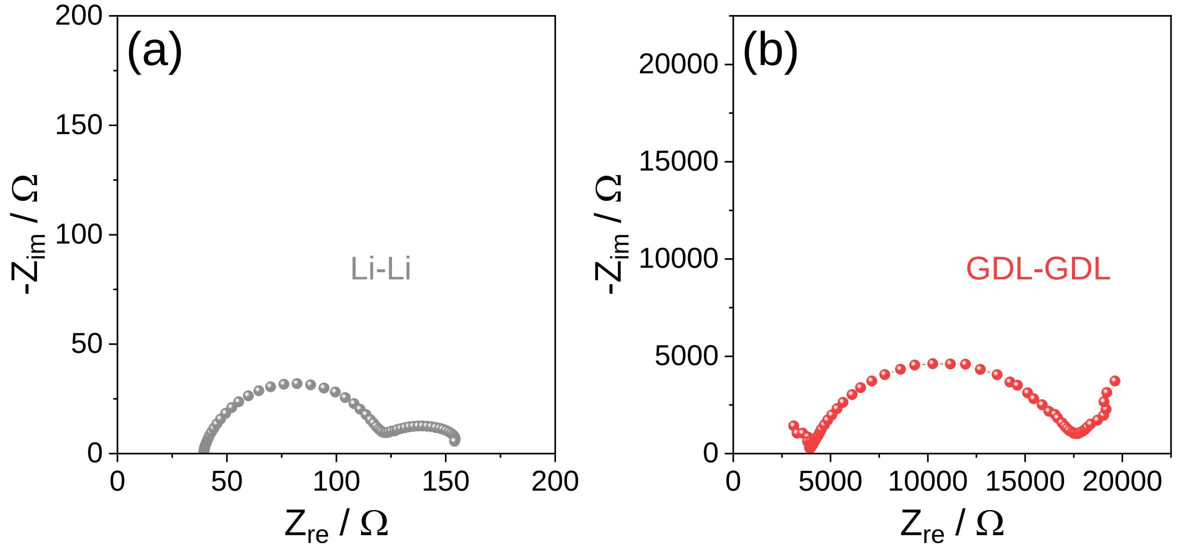


**Figure S5**. Nyquist plots recorded by EIS on (a) Li-Li (b) GDL-GDL cell in $O_2$ atmosphere at the OCV condition (see Experimental section in the Manuscript for cells configuration); GDL: 39BB; frequency range: 500 kHz – 100 mHz; alternate voltage signal: 10 mV.

Figure S6 reports the trends of the cell polarization at the end of charge/discharge processes, indicated as the overvoltage at the cell cutoffs, as a function of the cycle number. All the investigated cells exhibit a decrease of the overvoltage upon the electrochemical activation of the bare GDLs, used as cathode, occurring during the first galvanostatic charge/discharge cycle. The cell overvoltage then increases after 2-3 cycles for all the GDLs except for 39BB, for which the cell overvoltage starts to increase only after the 4$^{th}$ cycle.

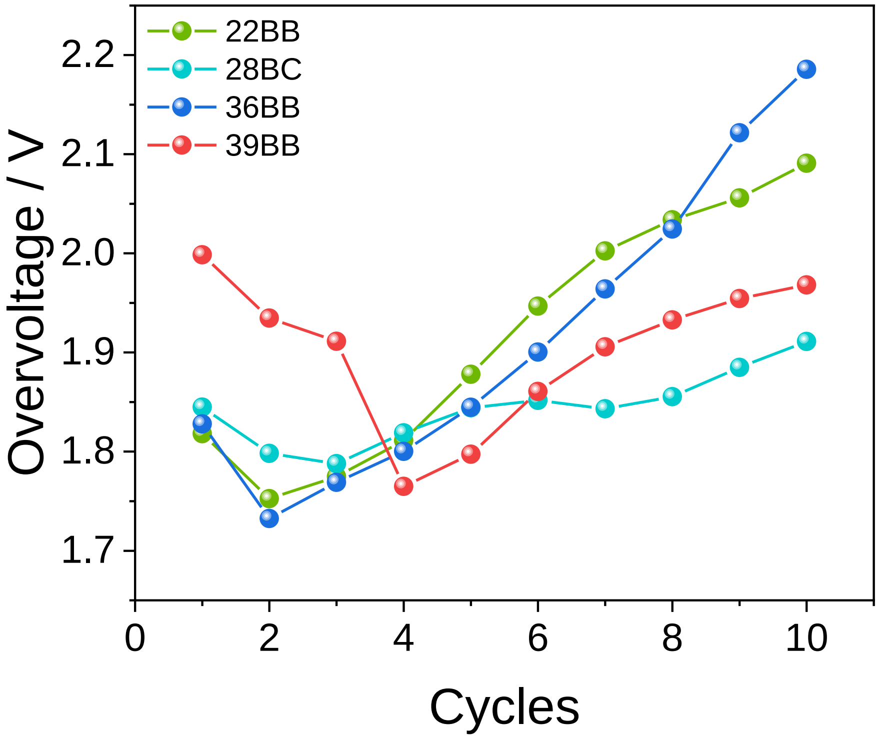


**Figure S6.** Overvoltage as a function of the galvanostatic charge/discharge cycles measured for the Li-$O_2$ cells using 22BB, 28BC, 36BB 39BB supports reported in Figure 4 of the Manuscript. The overvoltage is determined as the difference between the voltage of the cell at the end of charge and discharge.

Figure S7 shows the potential vs. time curves measured through GITT for the Li-$O_2$ cells depicted in Figure 6 of the Manuscript using 22BB (Figure S7a), 28BC (Figure S7b), 36BB (Figure S7c) and the 39BB (Figure S7d) as cathodes. The linear fitting of the relaxation potential vs. $t^{1/2}$ allows $dE/dt^{1/2}$ to be determined (see equation (1) in the Manuscript).[2,3]

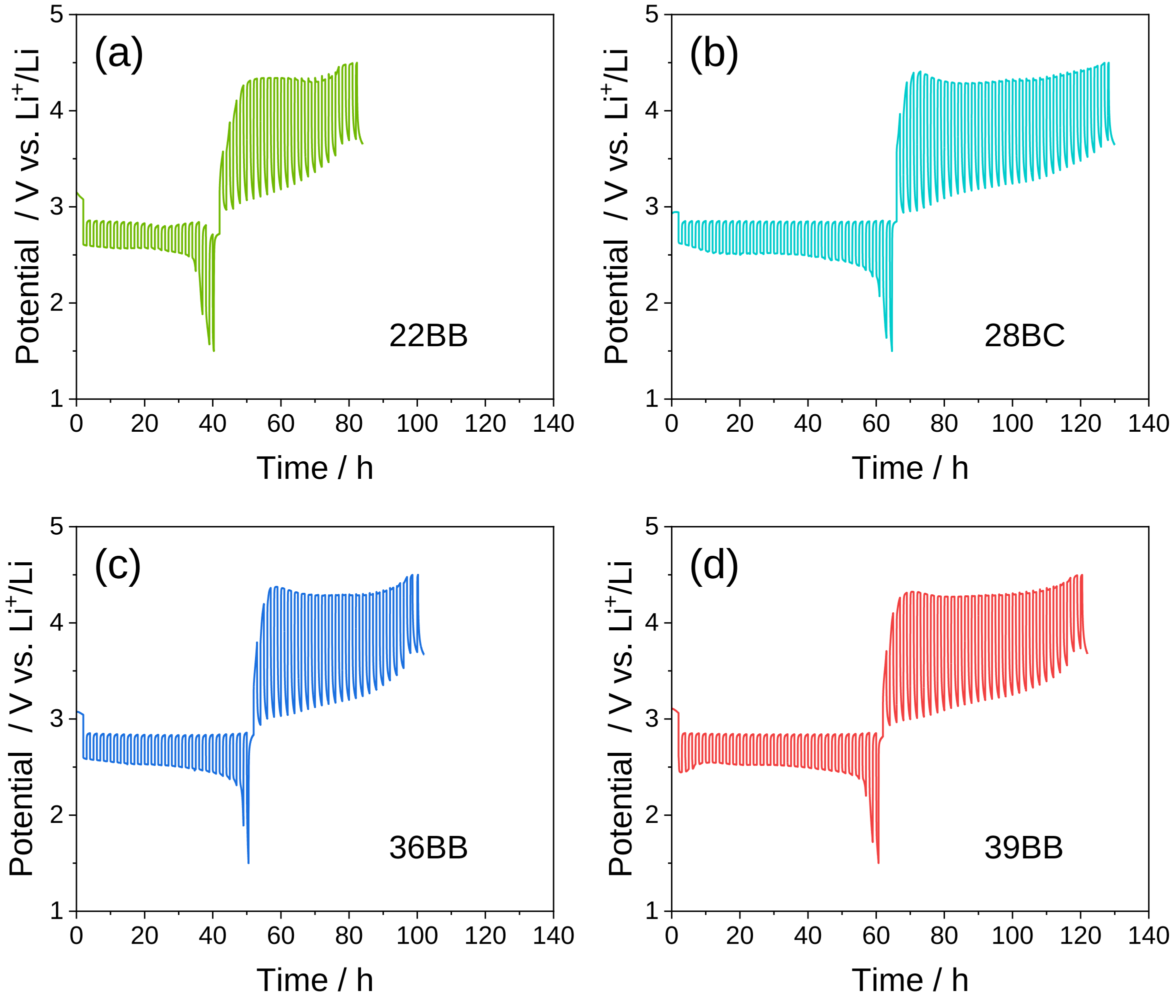


**Figure S7.** GITT curves (potential vs. time) measured for the Li-$O_2$ cells using **(a)** 22BB, **(b)** 28BC, **(c)** 36BB or **(d)** 39BB as the cathodes (see Figure 6 in the Manuscript).

**Table S1** Maximum and minimum *D* values calculated through GITT (see equation 1 in the Manuscript)[3] for Li-$O_2$ cells using the investigated GDLs as cathodes reported in Figure 6 of the Manuscript.

| GDL | Cell condition | $D_{max}$ [cm$^2$ s$^{-1}$] | $D_{min}$ [cm$^2$ s$^{-1}$] |
|---|---|---|---|
| 22BB | Discharge | $3.3\times10^{-9}$ | $4.5\times10^{-15}$ |
| | Charge | $8.8\times10^{-10}$ | $1.3\times10^{-16}$ |
| 28BC | Discharge | $2.8\times10^{-8}$ | $8.5\times10^{-14}$ |
| | Charge | $3.9\times10^{-10}$ | $4.4\times10^{-17}$ |
| 36BB | Discharge | $1.6\times10^{-8}$ | $1.1\times10^{-11}$ |
| | Charge | $6.4\times10^{-10}$ | $2.1\times10^{-16}$ |
| 39BB | Discharge | $9.1\times10^{-9}$ | $5.4\times10^{-14}$ |
| | Charge | $4.4\times10^{-10}$ | $1.1\times10^{-16}$ |

Figure S8 reports the polarization tests performed through galvanodynamic reduction scans on Li-Li or Li-GDL(39BB) cells in $O_2$ atmosphere. The data suggest a limiting current exceeding 5 mA $cm^{-2}$ for $Li^+$ diffusion in the Li-Li symmetrical system, and a double slope possibly including oxygen and lithium diffusion for the Li-GDL(39BB) cell.

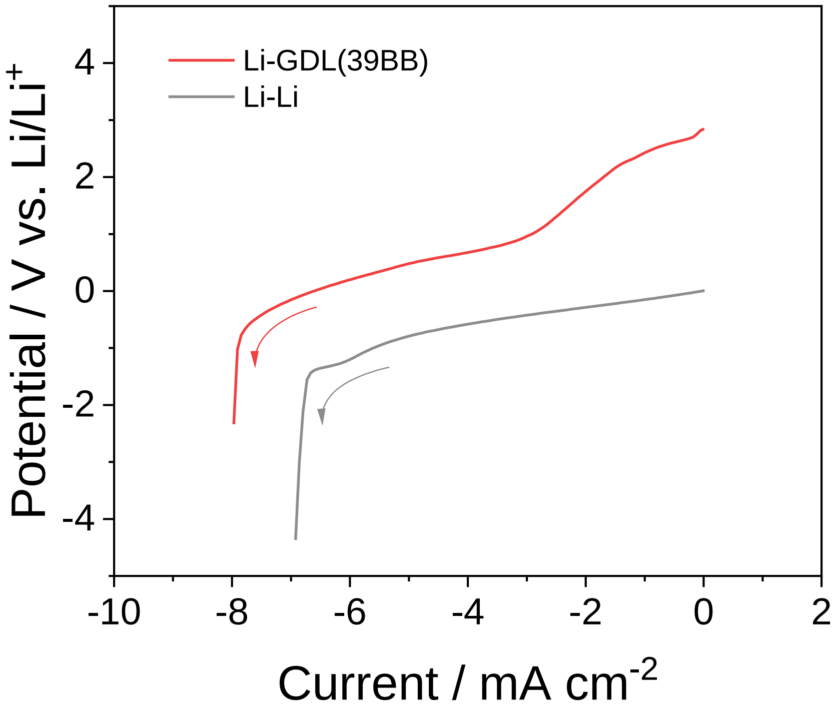


**Figure S8**. Polarization curves recorded through galvanodynamic reduction scans between 0 and -20 mA on either a Li-Li and Li-GDL(39BB) cells in $O_2$ atmosphere (see Experimental section in the Manuscript for set-up details); step height: 0.1 mA; step time: 10 s.

**Table S2**. Relative atomic concentrations of the elements detected on pristine and cycled electrodes measured by XPS in Figure 8 of the Manuscript.

| Element | Pristine electrode (at.%) | Cycled electrode (at.%) |
|---|---|---|
| **C** | 84.0 | 58.3 |
| **O** | 0.9 | 17.1 |
| **F** | 15.1 | 15.5 |
| **S** | - | 2.4 |
| **Li** | - | 6.7 |

**References**


(1) Nasef, M. M. Thermal Stability of Radiation Grafted PTFE-g-Polystyrene Sulfonic Acid Membranes. *Polym. Degrad. Stab.* **2000**, *68*, 231–238.

(2) Lama, F. L.; Marangon, V.; Caballero, Á.; Morales, J.; Hassoun, J. Diffusional Features of a Lithium-Sulfur Battery Exploiting Highly Microporous Activated Carbon. *ChemSusChem* **2023**, *16*, No. e202202095.

(3) Weppner, W.; Hugguns, R. A. Determination of the Kinetic Parameters of Mixed-Conducting Electrodes and Application to the System $Li_3Sb$. *J. Electrochem. Soc.* **1977**, *124*, 1569.
